# Design and Synthesis of Thiazolo[5,4-*f*]quinazolines as DYRK1A Inhibitors, Part II

**DOI:** 10.3390/molecules191015411

**Published:** 2014-09-26

**Authors:** Alicia Foucourt, Damien Hédou, Carole Dubouilh-Benard, Angélique Girard, Thierry Taverne, Anne-Sophie Casagrande, Laurent Désiré, Bertrand Leblond, Thierry Besson

**Affiliations:** 1 Normandie Univ, Laboratoire C.O.B.R.A., UMR 6014 and FR 3038; Univ Rouen; INSA de Rouen; CNRS, Bâtiment I.R.C.O.F. rue Tesnière, Mont-Saint-Aignan F-76821, France; E-Mails: foucourtalicia@aol.com (A.F.); damien.hedou@etu.univ-rouen.fr (D.H.); carole.dubouilh@univ-rouen.fr (C.D.-B.); 2 Diaxonhit, 65 boulevard Masséna, Paris F-75013, France; E-Mails: angelique.girard@diaxonhit.com (A.G.); taverne.thierry@gmail.com (T.T.); anne-sophie.casagrande@diaxonhit.com (A.-S.C.); laurent.desire@diaxonhit.com (L.D.); bertrandleblond@hotmail.com (B.L.)

**Keywords:** thiazolo[5,4-*f*]quinazolines, kinase inhibitors, DYRK1A, DYRK1B, microwave-assisted chemistry, Dimroth rearrangement, EHT 5372, EHT 6840, EHT 1610, EHT 9851, EHT 3356

## Abstract

The convenient synthesis of a focused library (forty molecules) of novel 6,6,5-tricyclic thiazolo[5,4-*f*]quinazolines was realized mainly under microwave irradiation. A novel 6-aminobenzo[*d*]thiazole-2,7-dicarbonitrile (**1**) was used as a versatile molecular platform for the synthesis of various derivatives. Kinase inhibition, of the obtained final compounds, was evaluated on a panel of two kinases (DYRK1A/1B) together with some known reference DYRK1A and DYRK1B inhibitors (harmine, TG003, NCGC-00189310 and leucettine L41). Compound IC_50_ values were obtained and compared. Five of the novel thiazolo[5,4-*f*]quinazoline derivatives prepared, EHT 5372 (**8c**), EHT 6840 (**8h**), EHT 1610 (**8i**), EHT 9851 (**8k**) and EHT 3356 (**9b**) displayed single-digit nanomolar or subnanomolar IC_50_ values and are among the most potent DYRK1A/1B inhibitors disclosed to date. DYRK1A/1B kinases are known to be involved in the regulation of various molecular pathways associated with oncology, neurodegenerative diseases (such as Alzeimer disease, AD, or other tauopathies), genetic diseases (such as Down Syndrome, DS), as well as diseases involved in abnormal pre-mRNA splicing. The compounds described in this communication constitute a highly potent set of novel molecular probes to evaluate the biology/pharmacology of DYR1A/1B in such diseases.

## 1. Introduction

Protein kinases catalyze protein phosphorylation, a key cellular regulatory mechanism, which is frequently dysregulated in human diseases. These enzymes are involved in all major diseases, including cancer, neurodegenerative disorders and cardiovascular diseases [1,2,3]. Consequently, protein kinases represent interesting targets for the pharmaceutical industry in its search for new therapeutic agents. Most kinases act on both serine and threonine, others act on tyrosine, and a number (dual-specificity kinases) act on all three. Our research groups are invested in the synthesis of polyaromatic heterocyclic molecules able to modulate the activity of kinases in signal transduction, and especially Ser/Thr kinases (CDK5, GSK3, CLK1 and CK1) and dual-specificity kinases (DYRK1A) [4,5,6,7,8], selected for their strong implication in various human pathologies, especially in AD [3].

**Scheme 1:**
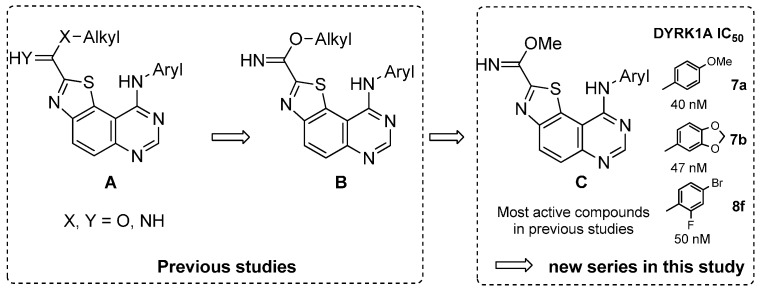
Structures of previous molecules (see part 1 [9]), which inspired the current work.

In the course of our work, the multistep synthesis of a novel 9-(aryl)-*N*-(2-alkyl)thiazolo[5,4-*f*]quinazoline library (**A** in Scheme 1) was recently described [9]. These compounds were designed as 6,6,5-tricyclic homologs of the basic 4-aminoquinazoline pharmacophore, which is present in approximately 80% of ATP-competitive kinase inhibitors that have received approval for the treatment of cancer [10]. Brief studies of their structure-activity relationships as kinase inhibitors were realized. Among the compounds tested, the most promising series (**B**) showed submicromolar activities against DYRK1A (0.04 μM < IC_50_ < 0.70 μM) and GSK3α/β kinases (0.16 μM < IC_50_ < 0.77 μM) with a marked preference for the first one [9]. Within this series, the DYRK1A IC_50_ values obtained for the three lead compounds (series **C** in Scheme 1: **7a**, **7b** and **8f** in this paper) were in the low nanomolar range (40 nM, 47 nM and 50 nM). This demonstrates that small sized groups linked to the thiazole moiety of the molecule were able to induce a strong enhancement of the inhibitory activity against DYRK1A. Interestingly, the three lead compounds possess a methylcarbimidate function in position 2 of the thiazole ring, associated with an *N*-aryl substituent on position 9 of the thiazolo[5,4-*f*]quinazoline scaffold (see compounds of the **C** series in Scheme 1).

The overall potential therapeutic interest of these compounds encouraged us to extend this series of thiazolo[5,4-*f*]quinazolines by substituting the position 4 of the pyrimidine ring with various aromatic amines and by introducing a methyl carbimidate group in position 2 of the thiazole moiety.

This paper describes the convenient preparation of a new methyl 9-(arylamino)thiazolo[5,4-*f*]quinazoline-2-carbimidate derivative library for which highly potent DYRK1A/1B kinase inhibitory activities are observed. The main part of the chemistry described was achieved under microwave irradiation as a continuation of our global strategy, which consists of designing adapted reactants and techniques offering operational, economic, and environmental benefits over conventional methods [11,12,13,14,15].

## 2. Results and Discussion

### 2.1. Synthesis 

**Scheme 2:**
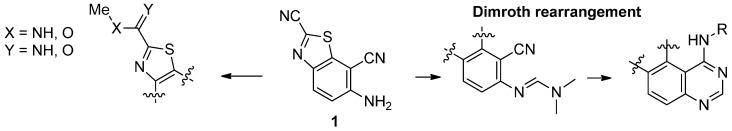
Envisioned transformations of **1** for synthesis of novel compounds of series **C**.

The target molecules we studied were thiazolo[5,4-*f*]quinazolines substituted in position 4 of the pyrimidine ring (which corresponds to position 9 of the tricyclic compound) by an aromatic amine. In order to have an efficient route to these various 9-anilinothiazolo[5,4-*f*]quinazolines, a rational multistep synthesis of a novel polyfunctionalized benzothiazole (see **1** in Scheme 2) has been performed [9]. This novel route was based on our previous Structure-Activity Relationship (SAR) studies conducted on the synthesis of such ring systems [12,13,14]. This molecular system was conceived to be an efficient precursor to various target molecules. On one side of compound **1** the presence of the versatile carbonitrile function in position 2 of the thiazole ring may allow easy access to a methylcarbimidate function. On the other side, the 2-aminobenzonitrile moiety offers a large panel of possibilities for extension of the aromatic structure by a heterocyclic core, such as pyrimidine (Scheme 2).

**Scheme 3:**
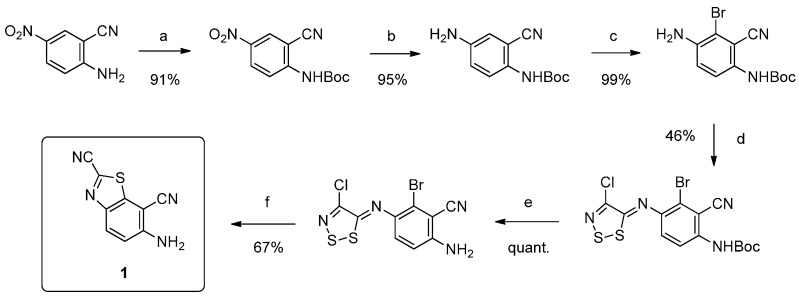
Multistep synthesis of the key benzothiazole **1**.

The synthesis of the key intermediate **1** was realized in six steps (overall yield of 23%) according to the described procedure [9] depicted in Scheme 3. Thus, ^2^*N*-protection of 2-aminobenzonitrile provided *tert*-butyl (2-cyano-4-nitrophenyl)carbamate, which was reduced by treatment with ammonium formate in the presence of a catalytic amount of 10% palladium charcoal. The resulting aromatic amine was selectively and quantitatively brominated in position 6 and the resulting *ortho*-bromo aniline was reacted with Appel salt (4,5-dichloro-1,2,3-dithiazolium chloride) to give an intermediate imino-1,2,3-dithiazole. The last intermediate was transformed into the final 6-aminobenzo[*d*]thiazole-2,7-dicarbonitrile (**1**) after ^2^*N*-deprotection and microwave-assisted copper-mediated cyclization. In terms of efficiency, 10 g of 2-amino-5-nitrobenzonitrile may lead to 2 g of polyfunctionalized benzo[*d*]thiazole **1**.

*Reagents and conditions*: (**a**) Boc_2_O, DMAP, Et_3_N, CH_2_Cl_2_, r.t., 4 h; (**b**) HCO_2_NH_4_, Pd.C, EtOH, 85 °C (μw), 30 min; (**c**) Br_2_, AcOH, CH_2_Cl_2_, r.t., 2.5 h; (**d**) Appel salt, Py. (2 eq), CH_2_Cl_2_, r.t., 4 h; (**e**) AcOH, 118 °C (μw), 2 h; (**f**) CuI, Py., 130 °C (μw), 20 min.

**Scheme 4:**
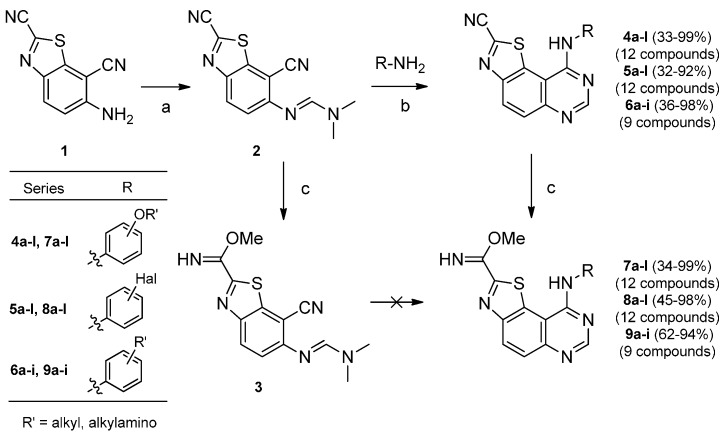
Synthesis of **7**, **8** and **9** series via transformation of **4**, **5** and **6** series.

The synthesis of the target molecules was continued by treatment of **1** with DMF/DMA under microwave irradiation at 70 °C to give (*E*)-*N*'-(2,7-dicyanobenzo[*d*]thiazol-6-yl)-*N*,*N*-dimethylformimidamide (**2**) in good yield (86%). At this stage of the synthesis, transformation of the carbonitrile group into methylcarbimidate was realized by microwave-assisted heating of **2** with sodium hydroxide (2.5 N in water) in methanol. Then, following a parallel chemistry strategy, the resulting product **3** was expected to serve as precursor for all the target molecules (e.g., **7a**–**l**, **8a**–**l** and **9a**–**i** in Scheme 4).

*Reagents and conditions*: (**a**) DMF/DMA, DMF, 70 °C (μw), 2 min, 86%; (**b**) aniline (1.5 equiv.), AcOH, 118 °C (μw), for time and yields see Table 1; (**c**) NaOMe (0.5 M in MeOH), MeOH, 65 °C (μw), 30 min, for yields see Table 1.

**Table 1:** Synthesis of carbonitrile **4**–**6** and their corresponding methyl carbimidates **7**–**9**.

Amine (R-NH_2_)	Compound	Yield (%) ^a^	Time (min)	Compound	Yield (%) ^a^
4-methoxyaniline	**4a**	99	2	**7a**	82
3,4-(methylenedioxy)aniline	**4b**	95	45	**7b**	92
1,4-benzodioxan-6-amine	**4c**	33	15	**7c**	80
2,3-dihydro-1-benzofuran-5-amine	**4d**	95	5	**7d**	66
3,4-dimethoxyaniline	**4e**	74	15	**7e**	89
2,4-dimethoxyaniline	**4f**	59	2	**7f**	71
3,5-dimethoxyaniline	**4g**	98	7	**7g**	58
3-nitro-4-methoxyaniline	**4h**	61	20	**7h**	59
4-aminophenol	**4i**	80	5	**7i**	81
5-amino-2-methoxyphenol	**4j**	54	5	**7j**	99
4-amino-2-nitrophenol	**4k**	60	15	**7k**	34
3,4,5-trimethoxyaniline	**4l**	85	5	**7l**	94
4-chloroaniline	**5a**	89	10	**8a**	62
3-chloroaniline	**5b**	74	20	**8b**	78
2,4-dichloroaniline	**5c**	32	50	**8c**	81
3,4-dichloroaniline	**5d**	42	20	**8d**	45
4-fluoroaniline	**5e**	92	5	**8e**	77
4-bromo-2-fluoroaniline	**5f**	78	30	**8f**	94
3-chloro-4-fluoroaniline	**5g**	82	10	**8g**	98
4-chloro-2-fluoroaniline	**5h**	56	20	**8h**	58
2-fluoro-4-methoxyaniline	**5i**	85	5	**8i**	82
4-amino-2-fluorophenol	**5j**	58	10	**8j**	70
2,4-difluoroaniline	**5k**	68	15	**8k**	71
4-aminobenzotrifluoride	**5l**	61	15	**8l**	53
aniline	**6a**	67	5	**9a**	52
4-toluidine	**6b**	64	2	**9b**	88
4- *tert*-butylaniline	**6c**	99	5	**9c**	69
3-ethynylaniline	**6d**	84	15	**9d**	68
4-aminobenzonitrile	**6e**	36	20	**9e**	52
3-aminobenzonitrile	**6f**	80	10	**9f**	33
6-aminobenzimidazole	**6g**	98	10	**9g**	57
*N*,*N*-dimethyl-*p*-phenylene-diamine	**6h**	25	15	**9h**	94
4-(pyrrolidin-1-yl)aniline	**6i**	48	5	**9i**	75

^a^ Isolated yields.

According to our previous experience, we envision to build the final molecule upon cyclization of the cyano pyridine 2 using a microwave-assisted thermal-sensitive Dimroth rearrangement [6] in which a nucleophilic attack of intermediate amidines by various aromatic amines would give the tricyclic products. Unfortunately, despite multiple attempts using several anilines and conditions, only degradation products were observed. This drawback incited us to envision the synthesis *via* a preliminary formation of the pyrimidine moiety before introducing the carbonitrile function. Although this route cannot be considered as the most efficient in terms of atoms involved and intermediates produced, it had the advantage of allowing the synthesis of the requested product-library. Then, cyclization of formimidamide **2** into thiazolo[5,4-*f*]quinazoline-2-carbonitriles **4a**–**l**, **5a**–**l** and **6a**–**i** was accomplished via thermal Dimroth rearrangement using 1.5 equivalents of the appropriate aniline in acetic acid under microwave irradiation at 118 °C for short periods of time. All the resulting compounds **4a**–**l**, **5a**–**l** and **6a**–**i** were obtained in good yields (see Table 1). The targeted methylcarbimidates **7a**–**l**, **8a**–**l** and **9a**–**i** were obtained in good to excellent yields (Scheme 4) after 30 min of microwave-assisted heating of compounds **4a**–**l**, **5a**–**l** and **6a**–**i** with a solution of sodium methoxide in methanol.

**Scheme 5:**
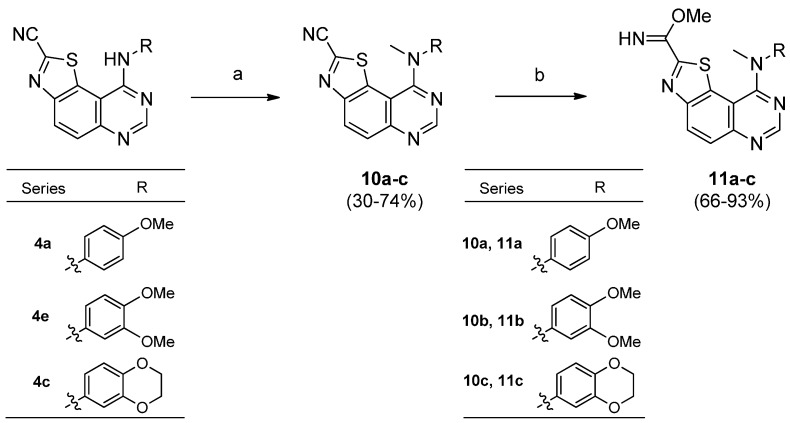
Synthesis of ^9^*N*-methylated derivatives of **7a**, **7c** and **7e**.

The presence of a secondary nitrogen atom in position 9 of the final skeleton may play an important role in the affinity shown by these compounds against DYRK1A. In order to confirm this hypothesis, and to understand the potential role of this amino group, the synthesis of ^9^*N*-methylated derivatives was undertaken. Three derivatives (**7a**, **7c** and **7e**) were chosen for their interesting IC_50_ values against DYK1A (Scheme 5).

*Reagents and conditions*: (**a**) ICH_3_, NaH, DMF, 0 °C then r.t., 2 h, 60% (**10a**); 74% (**10b**); 30% (**10c**); (**b**) NaOMe (0.5 M in MeOH), MeOH, 65 °C (μw), 30 min, 93% (**11a**); 73% (**11b**); 66% (**11c**).

First attempts consisting of direct *N*-methylation of compounds **7a**, **7c** and **7e** have not yielded to the desired products **11a**, **11b** and **11c** but generated various degradation products that were not isolated from the reaction medium. As an alternative, *N*-Methylation of the starting derivatives **4a**, **4c** and **4e** was succesfully realized by treatment with methyliodide and sodium hydride in DMF at room temperature. Compounds **10a**, **10b** and **10c** were then obtained from **4a**, **4e** and **4c** in 60, 74 and 30% yields, respectively. Transformation of their carbonitrile function in position 2 of the thiazole was performed in usual conditions to give the methyl carbimidate derivatives **11a**, **11b** and **11c** in good yields (66%–93%) (Scheme 5).

**Scheme 6:**
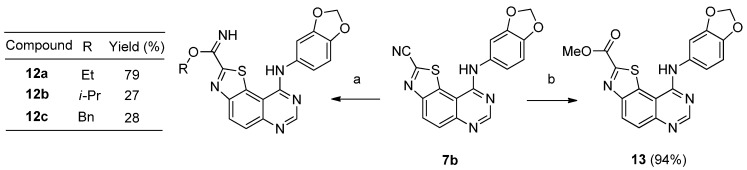
Synthesis of ethyl, isopropyl and benzyl carbimidates **12a**–**c** and methyl carboxylate **13** from carbonitrile **7b**.

In our previous work, the tricyclic derivatives **7b** was considered as one of the most active against DYRK1A (IC_50_ = 47 nM). The optimal size of the imidamide alkyl substituent seemed to be limited to one or two carbons. In order to define the real impact of this alkyl group in the activity of the molecule, a study consisting to extend its size was envisioned. Synthesis of a small library of various derivatives of **4b** was realized by heating this carbonitrile with various sodium alcoholates (ethylate, isopropylate and benzylate) in their corresponding alcohol (ethanol, isopropanol and benzylalcohol). Ethyl, isopropyl and benzyl carbimidates **12a**, **12b** and **12c** were obtained in convenient yields (79%, 27% and 28%, respectively) (Scheme 6). The parent compound **4b** was also transformed into its corresponding methyl ester **13** by treatment with a mixture of MeOH/H_2_O-TFA (0.1%) (6–4; v/v) at room temperature for 12 h and was obtained in excellent yield (94%) (Scheme 6).

Note that microwave heating was mainly performed at atmospheric pressure in a controlled multimode cavity with a microwave power delivery system ranging from 0 to 1200 W. Concerning the technical aspect, the choice of a reactor able to work at atmospheric pressure was guided by our previous experience in microwave-assisted heterocyclic synthesis, especially in the chemistry of quinazolines [6,7,8,9]. Open vessel microwave experiments have some advantages, such as the possibility of easier scale-up and the possibility to use current laboratory glassware. Our choice was also guided by a recent work describing the tendency of pressure to accumulate when a product as DMF/DMA was heated into pressurized vials, especially under microwaves [16]. In the main part of reactions studied, 600–800 W irradiation was enough to efficiently reach the programmed temperature. This parameter was mainly monitored via a contactless-infrared pyrometer, which was calibrated in control experiments with a fiber-optic contact thermometer.

*Reagents and conditions:* (**a**) RONa (0.5–1.0 M in ROH), ROH, 80–100 °C (μw), 30 min–2 h, R = Et (**12a**), *i*-Pr (**12b**) and Bn (**12c**); (**b**) MeOH-H_2_O/TFA (0.1%) (6:4, v/v), r.t., 12 h.

### 2.2. Biological Studies

DYRK1A kinase is a novel, high-potential therapeutic target for pharmacological interventions seeking to modify the course of AD [17,18,19]. The interest of our screening efforts was to discover new scaffolds able to inhibit efficiently DYRK1A. Before profiling our compound on a large kinase panel, we focussed our attention on DYRK1A and DYRK1B. This choice may be explained by the fact that the two amino acid sequences are 84% identical in the *N*-terminus and the catalytic domain [20]. 

The DYRK1A and DYRK1B kinase assays to determine IC_50_ values were performed by Reaction Biology Corporation using HotSpot technology (for a brief description see experimental section). Results are reported in Table 2.

**Scheme 7:**
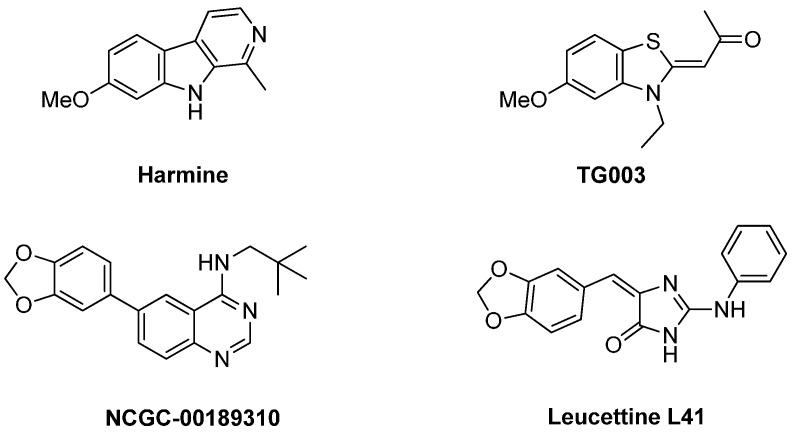
Structure of the DYRK1A/1B reference compounds used in this study.

Harmine [21,22], TG003 (*O*-methylated derivative of INDY) [23], NCGC-00189310 [24] and leucettine L41 [25,26] were also tested as reference DYRK1A and DYRK1B inhibitors (Scheme 7). These molecules, considered as reference tool compounds, were commercially available (harmine, TG003) or synthesized (NCGC-00189310, leucettine L41) to probe the activity at DYRK1A [20]. Their IC_50_ values were compared with those obtained for the compounds under study (see Table 2).

**Table 2:** DYRK1A and DYRK1B kinase inhibitory activity^a^ of the four methyl thiazolo[5,4-*f*]quinazoline carbimidate series (**7**, **8**, **9**, and **11**); ethyl, isopropyl and benzyl carbimidates (**12a**–**c**) and methyl carboxylate (**13**).

Amine in Position 9 (R-NH_2_)	Compound	DYRK1A IC_50_ (nM)	DYRK1B IC_50_ (nM)
4-methoxyaniline	**7a**	13.08 ^c^	19.22
3,4-(methylenedioxy)aniline	**7b**	1.65 ^c^	4.20
1,4-benzodioxan-6-amine	**7c**	8.00	17.60
2,3-dihydro-1-benzofuran-5-amine	**7d**	1 < IC_50_ < 1000	- ^b^
3,4-dimethoxyaniline	**7e**	128.80	160.6
2,4-dimethoxyaniline	**7f**	9.53	11.13
3,5-dimethoxyaniline	**7g**	298.90	530.90
3-nitro-4-methoxyaniline	**7h**	123.50	599.80
4-aminophenol	**7i**	1 < IC_50_ < 1000	- ^b^
5-amino-2-methoxyphenol	**7j**	1 < IC_50_ < 1000	- ^b^
4-amino-2-nitrophenol	**7k**	4.91	5.68
3,4,5-trimethoxyaniline	**7l**	436.10	485.80
4-chloroaniline	**8a**	1.13	4.74
3-chloroaniline	**8b**	13.64	18.78
2,4-dichloroaniline	**8c (EHT 5372)**	0.22	0.28
3,4-dichloroaniline	**8d**	66.82	99.34
4-fluoroaniline	**8e**	6.06	9.64
4-bromo-2-fluoroaniline	**8f**	3.6 ^c^	6.55
3-chloro-4-fluoroaniline	**8g**	1 < IC_50_ < 1000	- ^b^
4-chloro-2-fluoroaniline	**8h (EHT 6840)**	0.99	1.63
2-fluoro-4-methoxyaniline	**8i (EHT 1610)**	0.36	0.59
4-amino-2-fluorophenol	**8j**	8.63	11.00
2,4-difluoroaniline	**8k (EHT 9851)**	0.94	1.07
4-aminobenzotrifluoride	**8l**	54.84	186.40
aniline	**9a**	1.81	3.48
4-toluidine	**9b (EHT 3356)**	0.98	2.83
4- *tert*-butylaniline	**9c**	39.03	93.84
3-ethynylaniline	**9d**	40.76	46.29
4-aminobenzonitrile	**9e**	3.89	7.69
3-aminobenzonitrile	**9f**	42.70	71.98
6-aminobenzimidazole	**9g**	4.44	4.65
*N*,*N*-dimethyl-*p*-phenylene-diamine	**9h**	35.64	64.28
4-(pyrrolidin-1-yl)aniline	**9i**	n.t.^d^	n.t.
4-methoxyaniline	**11a**	79.85	84.94
3,4-dimethoxyaniline	**11b**	3768.00	4458.00
1,4-benzodioxan-6-amine	**11c**	1 < IC_50_ < 1000	- ^b^
3,4-(methylenedioxy)aniline	**12a**	6.02	7.72
3,4-(methylenedioxy)aniline	**12b**	124.7	217.80
3,4-(methylenedioxy)aniline	**12c**	33.93	37.34
3,4-(methylenedioxy)aniline	**13**	1 < IC_50_ < 1000	- ^b^
harmine		21.83	27.87
TG003		24.01	34.39
NCGC-00189310		2.20	20.57
leucettine L41		7.60	37.00

^a^ IC_50_ values are reported in nM. The most significant results are presented in bold; ^b^ Not determined; ^c^ Compared to our previous studies (see data given in Scheme 1 for **7a**, **7b** and **8f**) the Reaction Biology Corporation DYRK1A kinase assay was about ten times more sensitive and new values given for these three compounds were found closer to the nanomolar range; ^d^ Not tested.

The results shown in Table 2 demonstrated that the thiazolo[5,4-*f*]quinazoline derivatives of series **8** showed highly potent inhibitory activity against DYRK1A and DYRK1B. 

On a general aspect, compounds of series **7**, **9**, **11** and **12** were less active against DYRK1A when compared to series **8**, with the exception of some compounds (**7b**, **7c**, **7f**, **7g** and **7k**) of series **7** and compounds (EHT 3356 (**9b**) and **9c**) of series **9** for which nanomolar IC_50_ values were observed. One product of series **9** (EHT 3356 (**9b**)) exhibited a subnanomolar affinity against DYRK1A (IC_50_ = 0.98 nM) and was in the single-digit nanomolar range against DYRK1B (IC_50_ = 2.83 nM).

Among the compounds tested, series **8** was really promising, showing inhibitory activities in the subnanomolar range for DYRK1A (0.22 nM < IC_50_ < 0.99 nM) and in the single-digit nanomolar and subnanomolar range for DYRK1B (0.28 nM < IC_50_ < 1.63 nM).

**Scheme 8:**
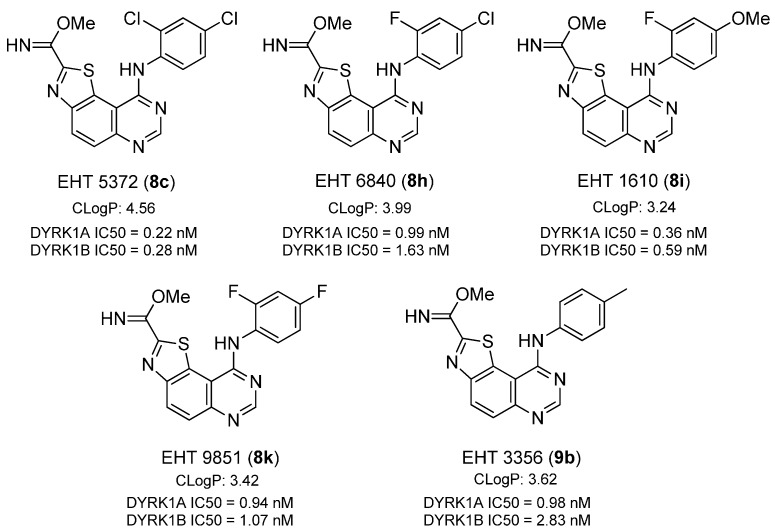
Structures and DYRK1A/1B IC_50_ values of the five lead compounds identified in this study (ClogP were calculated with Chemdraw V12.0).

The five most active molecules (Scheme 8) prepared in this study were EHT 5372 (**8c**), EHT 1610 (**8i**), EHT 9851 (**8k**), EHT 3356 (**9b**) and EHT 6840 (**8h**) which possess a phenyl group in ^9^*N* itself disubstituted in *ortho* and *para* by halogen atoms (Cl and F). An exception was made in the case of EHT 1610 (**8i**) in which a fluoride atom in *ortho* was accompanied by a methoxy group in *para* and in the case of EHT 3356 (**9b**) which only bears a methyl group in the *para* position.

We observed that the size and the position of the halogen atoms on the aniline in position 9 of the tricyclic skeleton seemed to have an important impact on the activity of the tested compounds. Considering the most active series **8**, molecules possessing substituents in the *meta* position (compared to the ^9^*N* nitrogen atom) showed lower affinity compared to their most active *ortho* and *para* disubstituted analogs.

Data given in Table 2 definitively confirmed that a methyl carbimidate substitutent in position 2 of the thiazole moiety of the molecules induced a high affinity for the DYRK1A/1B kinases. Given these findings, the second part of this exploratory work completes the observations published in the first study [9]. Looking at the results obtained for **7b** (DYRK1A IC_50_ = 1.65 nM) and its ethyl (**12a**: DYRK1A IC_50_ = 6.02 nM), isopropyl (**12b**: DYRK1A IC_50_ = 124.7 nM) and benzyl (**12c**: DYRK1A IC_50_ = 33.93 nM) carbimidate analogs clearly demonstrated that more than one carbon-size substituent on the oxygen atom of the imidate function led to a progressive decrease of affinity. This evolution in the IC_50_ values seemed to be linked to the steric hindrance, whilst the isopropyl group (see **12b**) was less active than the benzyl derivative (**12c**). The methyl carboxylate analog (see **13**) of methyl carbimidate **7b** was mostly inactive showing a dramatic decrease of its affinity for DYRK1A. This result needs to be confirmed but it seems to indicate that the imidate function was crucial for the inhibitory activity.

Another interesting result arose from series **11a**–**c** in which three compoundsbear a tertiary amine in postion 9 of the thiazoloquinazoline ring. *N*-Methylation of the nitrogen atom placed in the fourth position of the pyrimidine moiety induced a dramatic decrease in the affinity of the molecules obtained. This fact led us to believe that the presence of a secondary nitrogen atom in this position was crucial for the affinity of our molecules for the binding site of the enzyme.

Comparing the inhibitory potency of EHT 5372 (**8c**), EHT 1610 (**8i**), EHT 9851 (**8k**), EHT 3356 (**9b**) and EHT 6840 (**8h**) on DYRK1A/1B with that of some tool compounds currently described (harmine, TG003, NCGC-00189310 and leucettine L41), it can be easily observed that our five lead compounds were all much more potent on both kinases, in particular being more potent that NCGC-00189310 and leucettine L41, the two most active reference inhibitors tested in this screening campaign (Table 1).

These impressive results confirm that the thiazolo[5,4-*f*]quinazoline scaffold has a great potential in the development of novel and highly potent dual inhibitors of DYRK1A and DYRK1B kinases that are involved in many neurodegenerative diseases (AD and other tauopathies), in genetic disease (DS), in oncology, and in diseases involving abnormal pre-mRNA splicing [27,28].

The kinase selectivity profile of EHT 5372 (**8c**) has been performed and a high degree of selectivity for DYRK1A/1B and over 339 kinases was observed [29]. These results will be further discussed together with the potential of EHT 5372 (**8c**) to inhibit *in vitro* DYRK1A-induced Tau phosphorylation, Aβ production and Aβ effects on phospho-Tau. These findings demonstrate that this class of compounds warrants further investigation as a novel, high-potential therapy for AD and other tauopathies [30].

Recently a prominent tumor-promoting role for DYRK1A was demonstrated in acute megakaryoblastic leukemia in children with DS (DS-AMKL) [31]. The chromosome 21 kinase DYRK1A controls cell cycle exit and survival during lymphoid development and is a novel therapeutic target in acute lymphoblastic leukemia (ALL). Both B- and T-lineage ALL express high levels of DYRK1A relative to other tumor types. EHT 1610 (**8i**) has been shown to dose-dependently induce apoptosis in B- and T-ALL cell lines and primary human pediatric ALL samples. Moreover, EHT 1610 induced apoptosis of primary ALL cells that were resistant to cytarabine, suggesting that DYRK1A inhibitors may be used in combination with standard ALL therapies for refractory or relapsed cases. Together, this data establishes novel essential roles for DYRK1A in both normal and malignant lymphoid development and provide a rationale for the design of DYRK1A-targeted ALL therapies [32].

Interestingly, DYRK1B (also called Mirk) is an attractive oncological target that is highly expressed when cancer cells are quiescent and expressed at very low levels in normal cells [33]. Pharmacological DYRK1B inhibition would reduce the ability of cells to enter into quiescence and sensitize cancer cells to conventional chemotherapeutic agents. The five DYRK1B lead compounds described in this work have been characterized in various *in vitro* cellular studies and EHT 5372 has showed promising activities in patient-derived ovarian cancer ascites spheroids and *in vivo* activities in a Panc1 xenograft model without detectable toxicity in mice [34,35,36,37]. These results establish the usefulness of this class of inhibitors for targeting cancer cells with high DYRK1B kinase activity.

These multiple examples of biological activity in different therapeutic areas further highlighted the importance of the discovery of the presently described thiazolo[5,4-*f*]quinazoline lead DYRK1A/1B inhibitors and why their therapeutic potential warrants further investigation. 

## 3. Experimental Section

### 3.1. General Information

All reactions were carried out under an inert atmosphere of argon or nitrogen and monitored by thin-layer chromatography with silica gel 60 F254 pre-coated aluminum plates (0.25 mm). Visualization was performed with a UV light at 254 and 312 nm. Purifications were carried out on an Armen Instrument Spot 2 Flash System equipped with a dual UV-Vis spectrophotometer (200–600 nm), a fraction collector (192 tubes), a dual piston pump (1 to 250 mL/min, P_max_ = 50 bar/725 psi) allowing quaternary gradients and an additional inlet for air purge. Samples can be injected in liquid or solid phase. Purification was edited and monitored on an integrated panel PC with a touch screen controlled by Armen Glider Flash v3.1d software. Biotage SNAP flash chromatography cartridges (KP-Sil, normal phase, 10 to 340 g) were used for the purification process. Melting points of solid compounds were measured on a WME Köfler hot-stage with a precision of +/−2 °C and are uncorrected. IR spectra were recorded on a PerkinElmer Spectrum 100 Series FT-IR spectrometer. Liquids and solids were applied on the Single Reflection Attenuated Total Reflectance (ATR) Accessories. Absorption bands are given in cm^−1^.

^1^H, ^13^C-NMR spectra were recorded on a Brucker DXP 300 spectrometer at 300 and 75 MHz respectively. Abbreviations used for peak multiplicities are s: singlet, d: doublet, t: triplet, q: quadruplet and m: multiplet. Coupling constants *J* are in Hz and chemical shifts are given in ppm and calibrated with DMSO-*d*_6_ or CDCl_3_ (residual solvent signals). Mass spectra analysis was performed by the Mass Spectrometry Laboratory of the University of Rouen. Mass spectra (EI) were recorded with a Waters LCP 1er XR spectrometer.

Dichloromethane was distilled from CaH_2_ under argon. NBS was recrystallized in water. Other reagents and solvents were used as provided by commercial suppliers.

Appel salt was prepared according to literature procedure [16] by the addition of chloroacetonitrile (1 eq) to a solution of sulfur dichloride (5 eq) in dichloromethane (50 mL). Adogen™ (3–4 drops) was then added and the reaction was placed in a bowl of cold water. The mixture was left for 18 h without stirring under CaCl_2_ tube protection*:* The dark olive green solid was removed from the walls of the flask, filtered off under a blanket of argon, washed abundantly with dichloromethane and dried under vacuum for 2–3 h (average yield: 85%): mp 172–174 °C (dec); IR (nujol) cm^−1^ 1707, 1358s, 1280s, 1253, 1083, 917, 828s, and 605.

DYRK1A/1B reference compounds harmine and TG003 were purchased at Sigma-Aldrich. NCGC-00189310 and leucettine L41 were synthesized following the experimental procedures described in [24,25], respectively. 

Microwave experiments were conducted at atmospheric pressure in a commercial microwave reactors especially designed for synthetic chemistry. Time indicated in the various protocols is the time measured when the mixtures reached the programmed temperature after a ramp period of 2 min. RotoSYNTH™ (Milestone S.r.l. Italy) is a multimode cavity with a microwave power delivery system ranging from 0 to 1200 W. Open vessel experiments were carried out in round bottom flasks (from 25 mL to 4 L) fitted with a reflux condenser. The temperature was monitored via a contact-less infrared pyrometer (IRT) and fiber-optic contact thermometer (FO). Temperature, pressure and power profiles were edited and monitored through the EASY-Control software provided by the manufacturer. 

### 3.2. Synthesis

*6-Aminobenzo[d]thiazole-2,7-dicarbonitrile (**1**) and (E)-N'-(2,7-dicyanobenzo[d]thiazol-6-yl)-N,N-dimethylformimidamide* (**2**) were prepared and characterized following the procedure described in Reference [9]. 

*(E)-Methyl 7-cyano-6-([(dimethylamino)methylene]amino)benzo[d]thiazole-2-carbimidate* (**3**). A stirred mixture of carbonitrile **2** (0.17 mmol) and NaOH (2.5N sol., 50 μL) in methanol (2.5 mL) was heated under microwaves (1200 W) at 80 °C for 45 min. The solvent was removed *in vacuo* and the crude residue purified by flash chromatography (DCM-EtOAc, 9:1) to afford the imidate **3** as a yellow solid (0.032 g, 66% yield); mp = 163–165 °C. ^1^H-NMR (300 MHz, DMSO-*d*_6_) δ 9.34 (s, 1H, *NH*), 8.22 (m, 2H), 7.49 (d, 1H, *J* = 9.0 Hz), 3.92 (s, 3H), 3.13 (s, 3H), 3.05 (s, 3H) ); ^13^C-NMR (75 MHz, DMSO-*d*_6_) δ 159.2, 156.0, 155.8, 155.2, 146.8, 139.7, 128.8, 119.4, 116.8, 97.1, 54.1, 34.2; HRMS calcd for C_13_H_14_N_5_OS [M + H]^+^: 288.0919, found 288.0930.

#### 3.2.1. Synthesis of Thiazolo[5,4-*f*]quinazoline-2-carbonitriles **4a**–**l**, **5a**–**l** and **6a**–**i**

A mixture of (*E*)-*N*'-(2,7-dicyanobenzo[*d*]thiazol-6-yl)-*N*,*N*-dimethylformimidamide **2** (0.05 g, 0.19 mmol) and the appropriate amine (0.29 mmol, 1.5 equiv) in acetic acid (2 mL) was heated under microwaves (600 W) at 118 °C. On completion (followed by TLC), the reaction was cooled to ambient temperature. The solvent was removed *in vacuo* and the crude residue was purified by flash chromatography to afford the expected compounds **4a**–**k**, **5a**–**l** and **6a**–**h**.

Series **4a**–**k**: Compounds Bearing ^9^*N*-Phenyl Groups with Electron-Donating Substituents (e.g., OH, OR and Derivatives)

*9-(4-Methoxyphenylamino)thiazolo[5,4-f]quinazoline-2-carbonitrile (**4a**) and* *9-(benzo[d][1,3]dioxol-5-ylamino)thiazolo[5,4-f]quinazoline-2-carbonitrile* (**4b**) were synthesized in Reference [9].

*9-(2,3-Dihydrobenzo[b][1,4]dioxin-6-ylamino)thiazolo[5,4-f]quinazoline-2-carbonitrile* (**4c**). Prepared from **2** and 1,4-benzodioxan-6-amine. Flash chromatography eluent (DCM-EtOAc, 8:2). Yield: 33%; yellow solid; mp = 180–190 °C (dec). IR (cm^−1^) ν*_max_* 3055, 2978, 2932, 2875, 2230, 1709, 1638, 1609, 1578, 1496, 1460, 1376, 1299, 1281, 1239, 1200, 1151, 1063, 916, 885, 814. ^1^H-NMR (300 MHz, DMSO-*d*_6_) δ 8.37 (d, 1H, *J* = 8.4 Hz), 7.85 (m, 1H), 7.76 (m, 1H), 6.88 (d, 1H, *J* = 8.4 Hz), 6.56 (m, 2H), 4.25 (s, 4H). HRMS calcd for C_18_H_12_N_5_O_2_S [M + H]^+^: 362.0712, found 362.0696.

*9-(2,3-Dihydrobenzofuran-5-ylamino)thiazolo[5,4-f]quinazoline-2-carbonitrile* (**4d**). Prepared from **2** and 2,3-dihydro-1-benzofuran-5-amine. Flash chromatography eluent (EtOAc). Yield: 95%; yellow solid; mp = 216–218 °C. IR (cm^−1^) ν*_max_* 2894, 2853, 2228, 1643, 1609, 1579, 1484, 1467, 1376, 1353, 1306, 1269, 1219, 1192, 1164, 1123, 978, 941, 881, 814; ^1^H-NMR (300 MHz, DMSO-*d*_6_) δ 8.49 (d, 1H, *J* = 8.7 Hz), 8.10 (s, 1H), 7.75 (d, 1H, *J* = 8.7 Hz), 7.22 (m, 1H), 7.04 (s, 1H), 6.78 (m, 1H), 4.53 (t, 2H, *J* = 8.7 Hz), 3.20 (t, 1H, *J* = 8.7 Hz); HRMS calcd for C_18_H_12_N_5_OS [M + H]^+^: 346.0763, found 346.0762.

*9-(3,4-Dimethoxyphenylamino)thiazolo[5,4-f]quinazoline-2-carbonitrile* (**4e**). Prepared from **2** and 3,4-dimethoxyaniline. Flash chromatography eluent (DCM-EtOAc, 8:2). Yield: 74%; yellow solid; mp > 260 °C. IR (cm^−1^) ν*_max_* 3267, 2839, 2226, 1644, 1610, 1583, 1507, 1460, 1443, 1379, 1308, 1260, 1227, 1201, 1166, 1150, 1129, 1020, 967, 935, 861, 839; ^1^H-NMR (300 MHz, DMSO-*d*_6_) δ 8.34 (m, 1H), 7.79 (m, 1H), 7.71 (m, 1H), 6.90 (d, 1H, *J* = 8.1 Hz), 6.54 (m, 2H), 4.26 (s, 6H); HRMS calcd for C_18_H_14_N_5_O_2_S [M + H]^+^: 364.0868, found 364.0850.

*9-(2,4-Dimethoxyphenylamino)thiazolo[5,4-f]quinazoline-2-carbonitrile* (**4f**). Prepared from **2** and 2,4-dimethoxyaniline. Flash chromatography eluent (DCM-EtOAc, 5:5). Yield: 59%; orange solid; mp = 255–257 °C. IR (cm^−1^) ν*_max_* 3401, 3081, 2948, 2837, 2233, 1600, 1565, 1540, 1525, 1505, 1443, 1417, 1329, 1278, 1231, 1203, 1159, 1132, 1088, 1030, 959, 915; ^1^H-NMR (300 MHz, DMSO-*d*_6_) δ 8.51 (d, 1H, *J* = 9.0 Hz), 7.98 (s, 1H), 7.83 (d, 1H, *J* = 9.0 Hz), 6.95 (m, 1H), 6.67 (s, 1H), 6.58 (d, 1H, *J* = 9.0 Hz), 3.78 (s, 3H), 3.73 (s, 3H); HRMS calcd for C_18_H_14_N_5_O_2_S [M + H]^+^: 364.0868, found 364.0856.

*9-(3,5-Dimethoxyphenylamino)thiazolo[5,4-f]quinazoline-2-carbonitrile* (**4g**). Prepared from **2** and 3,5-dimethoxyaniline. Flash chromatography eluent (DCM-EtOAc, 5:5). Yield: 98%; yellow solid; mp = 248–250 °C. IR (cm^−1^) ν*_max_* 3242, 2940, 2837, 2223, 1711, 1647, 1578, 1455, 1419, 1383, 1357, 1306, 1265, 1205, 1144, 1058, 1046, 968, 943, 917, 833, 806; ^1^H-NMR (300 MHz, DMSO-*d*_6_) δ 8.51 (d, 1H, *J* = 9.0 Hz), 8.06 (s, 1H), 7.79 (d, 1H, *J* = 9.0 Hz), 6.31 (m, 3H), 3.74 (s, 6H); HRMS calcd for C_18_H_14_N_5_O_2_S [M + H]^+^: 364.0868, found 364.0856.

*9-(4-Methoxy-3-nitrophenylamino)thiazolo[5,4-f]quinazoline-2-carbonitrile* (**4h**). Prepared from **2** and 3-nitro-4-methoxyaniline. Flash chromatography eluent (DCM-EtOAc, 7:3). Yield: 61%; yellow solid, mp = 200–260 °C (dec). IR (cm^−1^) ν*_max_* 2226, 1644, 1523, 1459, 1346, 1267, 1191, 1155, 1075, 1015, 970, 928, 890, 822; ^1^H-NMR (300 MHz, DMSO-*d*_6_) δ 8.47 (d, 1H, *J* = 9.0 Hz), 8.26 (s, 1H), 7.97 (d, 1H, *J* = 9.0 Hz), 7.70 (s, 2H), 7.32 (d, 1H, *J* = 9.0 Hz), 3.93 (s, 3H); HRMS calcd for C_17_H_11_N_6_O_3_S [M + H]^+^: 379.0613, found 379.0614.

*9-(4-Hydroxyphenylamino)thiazolo[5,4-f]quinazoline-2-carbonitrile* (**4i**). Prepared from **2** and 4-aminophenol. Flash chromatography eluent (EtOAc). Yield: 80%; orange solid; mp = 236–238 °C. IR (cm^−1^) ν*_max_* 3072, 2225, 1641, 1615, 1577, 1503, 1464, 1378, 1350, 1307, 1230, 1212, 1159, 1097, 972, 832; ^1^H-NMR (300 MHz, DMSO-*d*_6_) δ 8.48 (d, 1H, *J* = 8.7 Hz), 8.08 (m, 1H), 7.76 (d, 1H, *J* = 8.7 Hz), 7.15 (m, 2H), 6.79 (m, 2H); HRMS calcd for C_16_H_10_N_5_OS [M + H]^+^: 320.0606, found 320.0619.

*9-(3-Hydroxy-4-methoxyphenylamino)thiazolo[5,4-f]quinazoline-2-carbonitrile* (**4j**). Prepared from **2** and 5-amino-2-methoxyphenol. Flash chromatography eluent (EtOAc). Yield: 54%; yellow solid; mp = 246–248 °C. IR (cm^−1^) ν*_max_* 2921, 2851, 2227, 1724, 1647, 1616, 1583, 1509, 1460, 1334, 1287, 1263, 1218, 1172, 1148, 1120, 1036, 973, 953, 864, 833; ^1^H-NMR (300 MHz, DMSO-*d*_6_) δ 8.49 (d, 1H, *J* = 8.7 Hz), 8.05 (m, 1H), 7.77 (d, 1H, *J* = 8.7 Hz), 7.94 (d, 2H, *J* = 8.7 Hz), 6.65 (m, 1H), 3.77 (s, 3H); HRMS calcd for C_17_H_12_N_5_O_2_S [M + H]^+^: 350.0712, found 350.0715.

*9-(4-Hydroxy-3-nitrophenylamino)thiazolo[5,4-f]quinazoline-2-carbonitrile* (**4k**). Prepared from **2** and 4-amino-2-nitrophenol. Flash chromatography eluent (DCM-EtOAc, 5:5). Yield: 60%; brown solid; mp > 260 °C. IR (cm^−1^) ν*_max_* 3334, 3081, 2926, 2225, 1627, 1591, 1569, 1525, 1465, 1419, 1395, 1305, 1237, 1171, 1132, 1070, 966, 930, 834, 819; ^1^H-NMR (300 MHz, DMSO-*d*_6_) δ 8.52 (d, 1H, *J* = 8.7 Hz), 8.28 (m, 1H), 8.03 (m, 1H), 7.75 (d, 1H, *J* = 8.7 Hz), 7.61 (m, 1H), 7.15 (d, 1H, *J* = 9.0 Hz); HRMS calcd for C_16_H_9_N_6_O_3_S [M + H]^+^: 365.0457, found 365.0441.

*9-(3,4,5-Trimethoxyphenylamino)thiazolo[5,4-f]quinazoline-2-carbonitrile* (**4l**). Prepared from **2** and 3,4,5-trimethoxyaniline. Yield: 94%; pale yellow solid; mp = 230–232 °C. IR (cm^−1^) ν*_max_* 3255, 3089, 3001, 2947, 2837, 2230, 1735, 1637, 1613, 1581, 1498, 1458, 1412, 1381, 1352, 1307, 1270, 1229, 1193, 1165, 1122, 1037, 1002, 991, 970, 952, 852, 830; ^1^H-NMR (300 MHz, DMSO-*d*_6_) δ 8.51 (d, 1H, *J* = 9.0 Hz), 8.05 (s, 1H), 7.80 (d, 1H, *J* = 9.0 Hz), 6.46 (s, 2H), 3.77 (s, 6H), 3.67 (s, 3H); HRMS calcd for C_19_H_16_N_5_O_3_S [M + H]^+^: 394.0974, found 394.0987.

Series **5a**–**l**: Compounds Bearing ^9^*N*-Phenyl Groups with Halogen Substituents (e.g., Cl, Br and F)

*9-(4-Chlorophenylamino)thiazolo[5,4-f]quinazoline-2-carbonitrile* (**5a**). Prepared from **2** and 4-chloroaniline. Flash chromatography eluent (DCM-EtOAc, 5:5). Yield: 89%; yellow solid; mp > 260 °C. IR (cm^−1^) ν*_max_* 2850, 2229, 1643, 1609, 1583, 1550, 1491, 1480, 1457, 1377, 1355, 1307, 1270, 1214, 1164, 1130, 1092, 1010, 980, 831; ^1^H-NMR (300 MHz, DMSO-*d*_6_) δ 8.51 (d, 1H, *J* = 8.7 Hz), 8.18 (s, 1H), 7.76 (d, 1H, *J* = 8.7 Hz), 7.38 (m, 2H), 7.04 (m, 2H); HRMS calcd for C_16_H_9_N_5_SCl [M + H]^+^: 338.0267, found 338.0274.

*9-(3-Chlorophenylamino)thiazolo[5,4-f]quinazoline-2-carbonitrile* (**5b**). Prepared from **2** and 3-chloroaniline. Flash chromatography eluent (DCM-EtOAc, 7:3). Yield: 74%; pale yellow solid; mp > 260 °C. IR (cm^−1^) ν*_max_* 2849, 2226, 1643, 1611, 1577, 1461, 1377, 1354, 1306, 1218, 1161, 1128, 1070, 974, 875, 833; ^1^H-NMR (300 MHz, DMSO-*d*_6_) δ 8.49 (d, 1H, *J* = 9.0 Hz), 8.21 (s, 1H), 7.74 (d, 1H, *J* = 9.0 Hz), 7.40–7.35 (m, 2H), 7.19–7.11 (m, 2H); HRMS calcd for C_16_H_9_N_5_SCl [M + H]^+^: 338.0267, found 338.0259.

*9-(2,4-Dichlorophenylamino)thiazolo[5,4-f]quinazoline-2-carbonitrile* (**5c**). Prepared from **2** and 2,4-dichloroaniline. Flash chromatography eluent (DCM-EtOAc, 5:5). Yield: 32%; yellow solid, mp = 260 °C. IR (cm^−1^) ν*_max_* 3063, 2231, 1736, 1644, 1611, 1577, 1459, 1380, 1355, 1310, 1242, 1173, 1098, 1051, 983, 830, 818; ^1^H-NMR (300 MHz, DMSO-*d*_6_) δ 8.56 (d, 1H, *J* = 9.0 Hz), 8.21 (s, 1H), 7.80 (d, 1H, *J* = 9.0 Hz), 7.63 (s, 1H), 7.39 (d, 1H, *J* = 8.1 Hz), 7.25 (d, 1H, *J* = 8.1 Hz); HRMS calcd for C_16_H_8_N_5_SCl_2_ [M + H]^+^: 371.9877, found 371.9877.

*9-(3,4-Dichlorophenylamino)thiazolo[5,4-f]quinazoline-2-carbonitrile* (**5d**). Prepared from **2** and 3,4-dichloroaniline. Flash chromatography eluent (DCM-EtOAc, 7:3). Yield: 42%; yellow solid; mp > 260 °C. IR (cm^−1^) ν*_max_* 2851, 2225, 1644, 1612, 1579, 1456, 1378, 1355, 1308, 1270, 1241, 1168, 1122, 1026, 971, 879, 834, 816; ^1^H-NMR (300 MHz, DMSO-*d*_6_) δ 8.55 (d, 1H, *J* = 8.7 Hz), 8.30 (s, 1H), 7.78 (d, 1H, *J* = 8.7 Hz), 7.63–7.53 (m, 2H), 7.30 (m, 2H); HRMS calcd for C_16_H_8_N_5_SCl_2_ [M + H]^+^: 371.9877, found 371.9882.

*9-(4-Fluorophenylamino)thiazolo[5,4-f]quinazoline-2-carbonitrile* (**5e**). Prepared from **2** and 4-fluoroaniline. Flash chromatography eluent (DCM-EtOAc, 5:5). Yield: 92%; yellow solid; mp > 260 °C. IR (cm^−1^) ν*_max_* 3049, 2840, 2226, 1722, 1643, 1610, 1581, 1557, 1502, 1377, 1355, 1305, 1269, 1227, 1208, 1166, 1130, 1090, 981, 846, 829, 818; ^19^F-NMR (282 MHz, DMSO-*d*_6_) δ −120.31; ^1^H-NMR (300 MHz, DMSO-*d*_6_) δ 8.51 (d, 1H, *J* = 9.0 Hz), 8.16 (s, 1H), 7.76 (d, 1H, *J* = 9.0 Hz), 7.26–7.08 (m, 4H); HRMS calcd for C_16_H_9_N_5_SF [M + H]^+^: 322.0563, found 322.0551.

*9-(4-Bromo-2-fluorophenylamino)thiazolo[5,4-f]quinazoline-2-carbonitrile* (**5f**) and 9-(3-chloro-4-fluorophenylamino)thiazolo[5,4-f]quinazoline-2-carbonitrile (**5g**) were synthesized in Reference [9].

*9-(4-Chloro-2-fluorophenylamino)thiazolo[5,4-f]quinazoline-2-carbonitrile* (**5h**). Prepared from **2** and 4-chloro-2-fluoroaniline. Flash chromatography eluent (DCM-EtOAc, 8:2). Yield: 56%; yellow solid, mp > 260 °C. IR (cm^−1^) ν*_max_* 2231, 1638, 1614, 1583, 1476, 1413, 1380, 1356, 1309, 1273, 1200, 1170, 1120, 982, 901, 838, 820; ^1^H-NMR (300 MHz, DMSO-*d*_6_) δ 8.55 (d, 1H, *J* = 9.0 Hz), 8.24 (s, 1H), 7.79 (d, 1H, *J* = 9.0 Hz), 7.45 (d, 1H, *J* = 9.0 Hz), 7.34 (t, 1H, *J* = 8.4 Hz), 7.26 (d, 1H, *J* = 9.0 Hz); HRMS calcd for C_16_H_8_N_5_SClF [M + H]^+^: 356.0173, found 356.0160.

*9-(2-Fluoro-4-methoxyphenylamino)thiazolo[5,4-f]quinazoline-2-carbonitrile* (**5i**). Prepared from **2** and 2-fluoro-4-methoxyaniline. Flash chromatography eluent (DCM-EtOAc, 5:5). Yield: 85%; yellow solid; mp > 260 °C. IR (cm^−1^) ν*_max_* 2844, 2226, 1731, 1649, 1613, 1583, 1507, 1493, 1460, 1445, 1379, 1356, 1305, 1263, 1212, 1168, 1153, 1129, 1090, 1027, 980, 947, 841, 830, 818; ^19^F-NMR (282 MHz, DMSO-*d*_6_) δ −120.02; ^1^H-NMR (300 MHz, DMSO-*d*_6_) δ 8.51 (d, 1H, *J* = 9.0 Hz), 8.15 (s, 1H), 7.77 (d, 1H, *J* = 9.0 Hz), 7.30 (s, 1H), 6.92 (m, 1H), 6.80 (d, 2H, *J* = 9.0 Hz); HRMS calcd for C_17_H_11_N_5_OSF [M + H]^+^: 352.0668, found 352.0658.

*9-(3-Fluoro-4-hydroxyphenylamino)thiazolo[5,4-f]quinazoline-2-carbonitrile* (**5j**). Prepared from **2** and 4-amino-2-fluorophenol. Flash chromatography eluent (DCM-EtOAc, 5:5). Yield: 58%; orange solid, mp > 260 °C. IR (cm^−1^) ν*_max_* 3375, 2228, 1731, 1649, 1619, 1578, 1512, 1470, 1373, 1347, 1292, 1241, 1204, 1150, 1111, 978, 943, 856, 836; ^19^F-NMR (282 MHz, DMSO-*d*_6_) δ −136.8; ^1^H-NMR (300 MHz, DMSO-*d*_6_) δ 8.46 (d, 1H, *J* = 9.0 Hz), 8.15 (s, 1H), 7.69 (d, 1H, *J* = 9.0 Hz), 6.97–6.81 (m, 3H); HRMS calcd for C_16_H_9_N_5_OSF [M + H]^+^: 338.0512, found 338.0516.

*9-(2,4-Difluorophenylamino)thiazolo[5,4-f]quinazoline-2-carbonitrile* (**5k**). Prepared from **2** and 2,4-difluoroaniline. Flash chromatography eluent (DCM-EtOAc, 7:3). Yield: 68%; yellow solid; mp > 260 °C. IR (cm^−1^) ν*_max_* 2228, 1645, 1611, 1583, 1557, 1488, 1460, 1378, 1357, 1311, 1276, 1260, 1172, 1138, 1091, 962, 854, 831, 818; ^19^F-NMR (282 MHz, DMSO-*d*_6_) δ −117.6, −118.7; ^1^H-NMR (300 MHz, DMSO-*d*_6_) δ 8.54 (d, 1H, *J* = 9.0 Hz), 8.22 (s, 1H), 7.78 (d, 1H, *J* = 9.0 Hz), 7.35–7.24 (m, 2H), 7.06 (t, 1H, *J* = 7.8 Hz); HRMS calcd for C_16_H_8_N_5_SF_2_ [M + H]^+^: 340.0468, found 340.0458.

*9-(4-(Trifluoromethyl)phenylamino)thiazolo[5,4-f]quinazoline-2-carbonitrile* (**5l**). Prepared from **2** and 4-aminobenzotrifluoride. Flash chromatography eluent (DCM-EtOAc, 7:3). Yield: 61%; yellow solid, mp > 260 °C. IR (cm^−1^) ν*_max_* 2851, 2229, 1649, 1604, 1582, 1512, 1457, 1382, 1318, 1272, 1252, 1221, 1165, 1117, 1101, 1062, 1011, 979, 863, 830; ^19^F-NMR (282 MHz, DMSO-*d*_6_) δ −60.01; ^1^H-NMR (300 MHz, DMSO-*d*_6_) δ 8.53 (d, 1H, *J* = 9.0 Hz), 8.22 (s, 1H), 7.77 (d, 1H, *J* = 9.0 Hz), 7.70 (d, 2H, *J* = 8.4 Hz), 7.39 (d, 2H, *J* = 8.4 Hz); HRMS calcd for C_17_H_9_N_5_SF_3_ [M + H]^+^: 372.0521, found 372.0531.

Series **6a**–**h**: Compounds Bearing ^9^*N*-Phenyl Groups with Alkyl, Amines and Nitrogen Containing Substituents

*9-(Phenylamino)thiazolo[5,4-f]quinazoline-2-carbonitrile* (**6a**). Prepared from **2** and aniline. Flash chromatography eluent (DCM-EtOAc, 5:5). Yield: 67%; yellow solid; mp > 260 °C. IR (cm^−1^) ν*_max_* 3395, 3057, 2228, 1731, 1644, 1608, 1577, 1491, 1459, 1378, 1352, 1301, 1255, 1214, 1147, 1128, 1106, 1071, 967, 896, 827; ^1^H-NMR (300 MHz, DMSO-*d*_6_) δ 8.51 (d, 1H, *J* = 9.0 Hz), 8.11 (s, 1H), 7.78 (d, 1H, *J* = 9.0 Hz), 7.40 (t, 2H, *J* = 7.5 Hz), 7.20 (m, 2H), 7.11 (t, 1H, *J* = 7.5 Hz); HRMS calcd for C_16_H_10_N_5_S [M + H]^+^: 304.0657, found 304.0657.

*9-(p-Tolylamino)thiazolo[5,4-f]quinazoline-2-carbonitrile* (**6b**). Prepared from **2** and 4-toluidine. Flash chromatography eluent (DCM-EtOAc, 7:3). Yield: 64%; yellow solid; mp = 260 °C. IR (cm^−1^) ν*_max_* 3016, 2853, 2228, 1731, 1641, 1605, 1581, 1554, 1505, 1458, 1376, 1353, 1304, 1268, 1215, 1165, 1130, 976, 831, 811; ^1^H-NMR (300 MHz, DMSO-*d*_6_) δ 8.49 (d, 1H, *J* = 9.0 Hz), 8.07 (s, 1H), 7.76 (d, 1H, *J* = 9.0 Hz), 7.20–7.17 (m, 2H), 7.12–7.05 (m, 2H), 2.32 (s, 3H); HRMS calcd for C_17_H_12_N_5_S [M + H]^+^: 318.0813, found 318.0811.

*9-(4-tert-Butylbenzylamino)thiazolo[5,4-f]quinazoline-2-carbonitrile* (**6c**). Prepared from **2** and 4-*tert*-butylaniline. Flash chromatography eluent (DCM-EtOAc, 7:3). Yield: 99%; yellow solid, mp = 154–156 °C. IR ((cm^−1^) ν*_max_* 2958, 2235, 1693, 1649, 1582, 1505, 1466, 1408, 1349, 1288, 1219, 1155, 1125, 989, 968, 894, 831; ^1^H-NMR (300 MHz, DMSO-*d*_6_) δ 8.49 (d, 1H, *J* = 9.0 Hz), 8.07 (s, 1H), 7.76 (d, 1H, *J* = 9.0 Hz), 7.40 (d, 2H, *J* = 7.8 Hz), 7.12 (m, 2H), 1.31 (s, 9H); HRMS calcd for C_20_H_18_N_5_S [M + H]^+^: 360.1283, found 360.1273.

*9-(3-Ethynylphenylamino)thiazolo[5,4-f]quinazoline-2-carbonitrile* (**6d**). Prepared from **2** and 3-ethynylaniline. Flash chromatography eluent (DCM-EtOAc, 7:3). Yield: 84%; yellow solid; mp = 182–184 °C. IR (cm^−1^) ν*_max_* 3295, 3062, 2846, 2225, 1642, 1612, 1581, 1566, 1458, 1404, 1376, 1348, 1306, 1263, 1229, 1165, 1148, 1126, 971, 910, 888, 835; ^1^H-NMR (300 MHz, DMSO-*d*_6_) δ 8.53 (d, 1H, *J* = 8.7 Hz), 8.20 (s, 1H), 7.78 (d, 1H, *J* = 8.7 Hz), 7.40–7.35 (m, 2H), 7.29 (m, 1H), 7.20 (m, 1H), 4.17 (s, 1H); HRMS calcd for C_18_H_10_N_5_S [M + H]^+^: 328.0657, found 328.0659.

*9-(4-Cyanophenylamino)thiazolo[5,4-f]quinazoline-2-carbonitrile* (**6e**). Prepared from **2** and 4-aminobenzonitrile. Flash chromatography eluent (DCM-EtOAc, 5:5). Yield: 36%; yellow solid; mp > 260 °C. IR (cm^−1^) ν*_max_* 3293, 2225, 2218, 1722, 1628, 1590, 1562, 1495, 1461, 1387, 1261, 1228, 1132, 966, 847, 814; ^1^H-NMR (300 MHz, DMSO-*d*_6_) δ 8.54 (d, 1H, *J* = 9.0 Hz), 8.28 (s, 1H), 7.77 (m, 3H), 7.40 (d, 2H, *J* = 9.0 Hz); HRMS calcd for C_17_H_9_N_6_S [M + H]^+^: 329.0609, found 329.0612.

*9-(3-Cyanophenylamino)thiazolo[5,4-f]quinazoline-2-carbonitrile* (**6f**). Prepared from **2** and 3-aminobenzonitrile. Yield: 40%; yellow solid, mp > 260 °C. IR (cm^−1^) ν*_max_* 3240, 3171, 3088, 2228, 1623, 1591, 1555, 1509, 1465, 1393, 1273, 1229, 1149, 969, 919, 825; ^1^H-NMR (300 MHz, DMSO-*d*_6_) δ 8.54 (d, 1H, *J* = 9.0 Hz), 8.43 (s, 1H), 7.74 (m, 2H), 7.55–7.53 (m, 3H); HRMS calcd for C_17_H_9_N_6_S [M + H]^+^: 329.0609, found 329.0600.

*9-(1H-Benzo[d]imidazol-6-ylamino)thiazolo[5,4-f]quinazoline-2-carbonitrile* (**6g**). Prepared from **2** and 6-aminobenzimidazole. Flash chromatography eluent (DCM-MeOH 8:2). Yield: 98%; yellow solid; mp > 260 °C. IR (cm^−1^) ν*_max_* 3084, 2226, 1615, 1557, 1464, 1376, 1347, 1248, 1147, 967, 939, 809; ^1^H-NMR (300 MHz, DMSO-*d*_6_) δ 8.48 (d, 1H, *J* = 8.7 Hz), 8.15–8.10 (m, 2H), 8.02 (m, 1H), 7.75 (d, 1H, *J* = 8.7 Hz), 7.56 (m, 1H), 7.04 (m, 1H); HRMS calcd for C_17_H_10_N_7_S [M + H]^+^: 344.0718, found 344.0705.

*9*-[4-(Dimethylamino)phenylamino]*thiazolo[5,4-f]quinazoline-2-carbonitrile* (**6h**). Prepared from **2** and *N*,*N*-dimethyl-*p*-phenylene-diamine. Flash chromatography eluent (DCM-EtOAc, 8:2). Yield: 25%; yellow solid; mp > 260 °C. IR (cm^−1^) ν*_max_* 3293, 2228, 1609, 1572, 1523, 1460, 1368, 1274, 1229, 1204, 1188, 1163, 1141, 1058, 1009, 948, 842, 811; ^1^H-NMR (300 MHz, DMSO-*d*_6_) δ 8.52 (d, 1H, *J* = 9.0 Hz), 8.16 (s, 1H), 7.86 (d, 1H, *J* = 9.0 Hz), 7.37 (d, 2H, *J* = 8.7 Hz), 6.92 (d, 2H, *J* = 8.7 Hz), 3.00 (s, 6H); HRMS calcd for C_18_H_15_N_6_S [M + H]^+^: 347.1079, found 347.1066.

*9*-[4-(Pyrrolidin-1-yl)phenylamino]*thiazolo[5,4-f]quinazoline-2-carbonitrile* (**6i**). Prepared from **2** and 4-(pyrrolidin-1-yl)aniline. Flash chromatography eluent (DCM-EtOAc, 8:2). Yield: 48%; yellow solid; mp > 260 °C. IR (cm^−1^) ν*_max_* 3303, 2842, 2233, 1709, 1629, 1613, 1583, 1522, 1466, 1388, 1347, 1275, 1219, 1185, 1166, 1060, 1014, 989, 828, 809; ^1^H-NMR (300 MHz, DMSO-*d*_6_) δ 8.54 (d, 1H, *J* = 9.0 Hz), 8.15 (s, 1H), 7.88 (d, 1H, *J* = 9.0 Hz), 7.35 (d, 2H, *J* = 8.1 Hz), 6.73 (d, 2H, *J* = 8.1 Hz), 3.17 (m, 4H), 1.99 (m, 4H); HRMS calcd for C_20_H_17_N_6_S [M + H]^+^: 373.1235, found 373.1218.

#### 3.2.2. Synthesis of Methyl Imidates **7a**–**l**, **8a**–**l** and **9a**–**i**

General procedure: a stirred mixture of carbonitriles **4a**–**l**, **5a**–**l** and **6a**–**i** (0.13 mmol) and NaOCH_3_ (0.5 M sol. in MeOH, 130 μL) in methanol (4 mL) was heated under microwaves at 65 °C (600W) for 30 min. The solvent was removed *in vacuo* and the crude residue purified by flash chromatography (DCM-EtOAc) to afford imidates **7a**–**l**, **8a**–**l** and **9a**–**i**.

Series **7a**–**l**: Compounds Bearing ^9^*N*-Phenyl Groups with Electron-Donating Substituents (e.g., OH, OR and Derivatives)

*Methyl 9-(4-methoxyphenylamino)thiazolo[5,4-f]quinazoline-2-carbimidate* (**7a**) and *methyl 9-(benzo[d][1,3]dioxol-5-ylamino)thiazolo[5,4-f]quinazoline-2-carbimidate* (**7b**) were synthesized in Reference [9].

*Methyl 9-(2,3-dihydrobenzo[b][1,4]dioxin-6-ylamino)thiazolo[5,4-f]quinazoline-2-carbimidate* (**7c**). Prepared from carbonitrile **4c**. Flash chromatography eluent (EtOAc). Yield: 80%; yellow solid; mp = 192–194 °C. IR (cm^−1^) ν*_max_* 3575, 3063, 1647, 1578, 1499, 1439, 1347, 1302, 1241, 1199, 1156, 1122, 1062, 948, 915, 836; ^1^H-NMR (300 MHz, DMSO-*d*_6_) δ 8.40 (d, 1H, *J* = 9 Hz), 8.01 (s, 1H), 7.69 (d, 1H, *J* = 9 Hz), 6.87 (d, 1H, *J* = 2.1 Hz), 6.67 (m, 2H), 4.24 (s, 4H), 3.94 (s, 3H); HRMS calcd for C_19_H_16_N_5_O_3_S [M + H]^+^: 394.0974, found 394.0954.

*Methyl 9-(2,3-dihydrobenzofuran-5-ylamino)thiazolo[5,4-f]quinazoline-2-carbimidate* (**7d**). Prepared from carbonitrile **4d**. Flash chromatography eluent (EtOAc). Yield: 66%; yellow solid; mp = 200–202 °C. IR (cm^−1^) ν*_max_* 3291, 3053, 2911, 1641, 1611, 1573, 1508, 1482, 1437, 1355, 1333, 1287, 1226, 1196, 1158, 1092, 1067, 985, 942, 821; ^1^H-NMR (300 MHz, DMSO-*d*_6_) δ 8.39 (d, 1H, *J* = 9 Hz), 8.01 (s, 1H), 7.69 (d, 1H, *J* = 9 Hz), 7.12 (m, 1H), 6.92 (m, 1H), 6.78–6.73 (m, 1H), 4.53 (t, 2H, *J* = 8.7 Hz), 3.95 (s, 3H), 3.19 (t, 2H, *J* = 8.7 Hz); HRMS calcd for C_19_H_16_N_5_O_2_S [M + H]^+^: 378.1025, found 378.1006.

*Methyl 9-(3,4-dimethoxyphenylamino)thiazolo[5,4-f]quinazoline-2-carbimidate* (**7e**). Prepared from carbonitrile **4e**. Flash chromatography eluent (EtOAc). Yield: 89%; yellow solid; mp = 216–218 °C. IR (cm^−1^) ν*_max_* 3289, 2921, 2852, 1651, 1613, 1583, 1505, 1466, 1432, 1376, 1348, 1309, 1261, 1226, 1195, 1164, 1146, 1128, 1075, 1027, 968, 945, 924, 854, 832; ^1^H-NMR (300 MHz, DMSO-*d*_6_) δ 8.44 (d, 1H, *J* = 9 Hz), 7.92 (s, 1H), 7.72 (d, 1H, *J* = 9 Hz), 6.99 (d, 1H, *J* = 2.1 Hz), 6.83 (d, 1H, *J* = 8.4 Hz), 6.75 (dd, 1H, *J*_1_ = 2.1 Hz, *J*_2_ = 8.4 Hz), 3.94 (s, 3H), 3.76 (s, 6H); HRMS calcd for C_19_H_18_N_5_O_3_S [M + H]^+^: 396.1130, found 396.1119.

*Methyl 9-(2,4-dimethoxyphenylamino)thiazolo[5,4-f]quinazoline-2-carbimidate* (**7f**). Prepared from carbonitrile **4f**. Flash chromatography eluent (EtOAc). Yield: 71%; pale green solid; mp = 244–246 °C. IR (cm^−1^) ν*_max_* 3380, 3277, 2999, 2942, 2828, 1654, 1608, 1566, 1545, 1526, 1506, 1455, 1431, 1332, 1276, 1204, 1152, 1123, 1097, 1063, 1026, 993, 963, 942, 916; ^1^H-NMR (300 MHz, DMSO-*d*_6_) δ 9.33 (s, 1H, NH), 8.41 (d, 1H, *J* = 9.0 Hz), 7.84 (s, 1H), 7.73 (d, 1H, *J* = 9.0 Hz), 6.88 (m, 1H), 6.68 (m, 1H), 6.58 (d, 1H, *J* = 7.8 Hz), 3.94 (s, 3H), 3.79 (s, 3H), 3.72 (s, 3H); HRMS calcd for C_19_H_18_N_5_O_3_S [M + H]^+^: 396.1130, found 396.1124.

*Methyl 9-(3,5-dimethoxyphenylamino)thiazolo[5,4-f]quinazoline-2-carbimidate* (**7g**). Prepared from carbonitrile **4g**. Flash chromatography eluent (EtOAc). Yield: 58%; pale yellow solid; mp = 258–260 °C. IR (cm^−1^) ν*_max_* 3237, 2955, 2929, 1731, 1660, 1579, 1495, 1440, 1368, 1347, 1301, 1250, 1189, 1149, 1107, 1058, 973, 953, 856, 824; ^1^H-NMR (300 MHz, DMSO-*d*_6_) δ 9.34 (s, 1H, NH), 8.43 (d, 1H, *J* = 9.0 Hz), 7.96 (s, 1H), 7.74 (d, 1H, *J* = 9.0 Hz), 6.25 (m, 3H), 3.94 (s, 3H), 3.74 (s, 6H); HRMS calcd for C_19_H_18_N_5_O_3_S [M + H]^+^: 396.1130, found 396.1128.

*Methyl 9-(4-methoxy-3-nitrophenylamino)thiazolo[5,4-f]quinazoline-2-carbimidate* (**7h**). Prepared from carbonitrile 153. Flash chromatography eluent (DCM-MeOH, 95:5). Yield: 59%; yellow solid; mp = 212–214 °C. IR (cm^−1^) ν*_max_* 1731, 1643, 1603, 1520, 1489, 1438, 1345, 1266, 1158, 1072, 1014, 946, 870, 821, 810; ^1^H-NMR (300 MHz, DMSO-*d*_6_) δ 9.32 (s, 1H, NH), 8.40 (d, 1H, *J* = 9.0 Hz), 8.17 (s, 1H), 7.82 (d, 1H, *J* = 9.0 Hz), 7.66 (d, 1H, *J* = 9.0 Hz), 7.55 (d, 1H, *J* = 8.1 Hz), 7.36 (d, 1H, *J* = 9.0 Hz), 3.95 (s, 3H), 3.92 (s, 3H); HRMS calcd for C_18_H_15_N_6_O_4_S [M + H]^+^: 411.0876, found 411.0869.

*Methyl 9-(4-hydroxyphenylamino)thiazolo[5,4-f]quinazoline-2-carbimidate* (**7i**). Prepared from carbonitrile **4i**. Flash chromatography eluent (EtOAc). Yield: 81%; yellow solid; mp = 194–196 °C. IR (cm^−1^) ν*_max_* 2953, 2852, 1644, 1619, 1573, 1508, 1477, 1372, 1326, 1235, 1164, 1100, 1077, 968, 940, 835; ^1^H-NMR (300 MHz, DMSO-*d*_6_) δ 8.38 (d, 1H, *J* = 9 Hz), 8.02 (s, 1H), 7.69 (d, 1H, *J* = 9 Hz), 7.04 (m, 2H), 6.80–6.73 (m, 2H), 3.94 (s, 3H); HRMS calcd for C_17_H_14_N_5_O_2_S [M + H]^+^: 352.0868, found 352.0873.

*Methyl 9-(3-hydroxy-4-methoxyphenylamino)thiazolo[5,4-f]quinazoline-2-carbimidate* (**7j**). Prepared from carbonitrile **4j**. Flash chromatography eluent (EtOAc). Quantitative yield; yellow solid; mp = 216–218 °C. IR (cm^−1^) ν*_max_* 3289, 2921, 2852, 1643, 1611, 1578, 1505, 1441, 1379, 1348, 1281, 1245, 1154, 1128, 1077, 1027, 957, 834; ^1^H-NMR (300 MHz, DMSO-*d*_6_) δ 8.40 (d, 1H, *J* = 9 Hz), 7.99 (s, 1H), 7.71 (d, 1H, *J* = 9 Hz), 6.94 (d, 1H, *J* = 8.4 Hz), 6.65–6.55 (m, 2H), 3.94 (s, 3H), 3.77 (s, 3H); HRMS calcd for C_18_H_16_N_5_O_3_S [M + H]^+^: 382.0974, found 382.0957.

*Methyl 9-(4-hydroxy-3-nitrophenylamino)thiazolo[5,4-f]quinazoline-2-carbimidate* (**7k**). Prepared from carbonitrile **4k**. Flash chromatography eluent (EtOAc). Yield: 34%; orange solid; mp = 208–201 °C. IR (cm^−1^) ν*_max_* 2957, 2911, 1724, 1622, 1560, 1520, 1476, 1379, 1310, 1243, 1156, 1070, 971, 945, 820; ^1^H-NMR (300 MHz, DMSO-*d*_6_) δ 8.30 (d, 1H, *J* = 8.7 Hz), 8.18 (m, 1H), 7.67 (m, 2H), 7.16 (d, 1H, *J* = 8.7 Hz), 3.96 (s, 3H); HRMS calcd for C_17_H_13_N_6_O_4_S [M + H]^+^: 397.0719, found 397.0710.

*Methyl 9-(3,4,5-trimethoxyphenylamino)thiazolo[5,4-f]quinazoline-2-carbimidate* (**7l**). Prepared from carbonitrile **4l**. Flash chromatography eluent (EtOAc). Yield: 87%; yellow solid; mp = 252–254 °C. IR (cm^−1^) ν*_max_* 3291, 2941, 2833, 1640, 1583, 1496, 1434, 1415, 1337, 1228, 1164, 1143, 1116, 1073, 993, 975, 954, 861, 844, 822; ^1^H-NMR (300 MHz, DMSO-*d*_6_) δ 9.34 (s, 1H, NH), 8.42 (d, 1H, *J* = 9.0 Hz), 7.94 (s, 1H), 7.74 (d, 1H, *J* = 9.0 Hz), 6.37 (s, 2H), 3.94 (s, 3H), 3.77 (s, 6H), 3.67 (s, 3H); HRMS calcd for C_20_H_20_N_5_O_4_S [M + H]^+^: 426.1236, found 426.1240.

Series **8a**–**l**: Compounds Bearing ^9^*N*-Phenyl Groups with Halogen Substituents (e.g., Cl, Br and F)

*Methyl 9-(4-chlorophenylamino)thiazolo[5,4-f]quinazoline-2-carbimidate* (**8a**). Prepared from carbonitrile **5a**. Flash chromatography eluent (EtOAc). Yield: 62%; yellow solid; mp > 260 °C. IR (cm^−1^) ν*_max_* 2948, 1644, 1604, 1557, 1509, 1481, 1435, 1401, 1356, 1285, 1240, 1159, 1094, 1074, 992, 943, 816; ^1^H-NMR (300 MHz, DMSO-*d*_6_) δ 8.42 (d, 1H, *J* = 9 Hz), 8.08 (s, 1H), 7.70 (d, 1H, *J* = 9 Hz), 7.41 (d, 2H, *J* = 8.1 Hz), 7.20 (m, 2H), 3.95 (s, 3H); HRMS calcd for C_17_H_13_N_5_OSCl [M + H]^+^: 370.0529, found 370.0521.

*Methyl 9-(3-chlorophenylamino)thiazolo[5,4-f]quinazoline-2-carbimidate* (**8b**). Prepared from carbonitrile **5b**. Flash chromatography eluent (EtOAc). Yield: 78%; pale yellow solid; mp = 234–236 °C. IR (cm^−1^) ν*_max_* 3293, 2950, 1639, 1593, 1550, 1507, 1470, 1437, 1355, 1286, 1157, 1070, 994, 968, 943, 876, 821; ^1^H-NMR (300 MHz, DMSO-*d*_6_) δ 9.32 (s, 1H, NH), 8.40 (d, 1H, *J* = 9.0 Hz), 8.11 (s, 1H), 7.67 (d, 1H, *J* = 9.0 Hz), 7.37 (t, 1H, *J* = 7.8 Hz), 7.23 (m, 1H), 7.12–7.08 (m, 2H), 3.94 (s, 3H); HRMS calcd for C_17_H_13_N_5_OSCl [M + H]^+^: 370.0529, found 370.0524.

*Methyl 9-(2,4-dichlorophenylamino)thiazolo[5,4-f]quinazoline-2-carbimidate* (**8c**). Prepared from carbonitrile **5c**. Flash chromatography eluent (EtOAc). Yield: 81%; yellow solid; mp > 260 °C. IR (cm^−1^) ν*_max_* 2953, 1727, 1641, 1586, 1507, 1488, 1464, 1394, 1354, 1284, 1158, 1099, 1073, 1054, 941, 860, 816; ^1^H-NMR (300 MHz, DMSO-*d*_6_) δ 9.34 (s, 1H, NH), 8.45 (d, 1H, *J* = 9.0 Hz), 8.10 (s, 1H), 7.71 (d, 1H, *J* = 9.0 Hz), 7.62 (s, 1H), 7.38 (d, 1H, *J* = 8.1 Hz), 7.18 (d, 1H, *J* = 8.1 Hz), 3.93 (s, 3H); HRMS calcd for C_17_H_12_N_5_OSCl_2_ [M + H]^+^: 404.0140, found 404.0146.

*Methyl 9-(3,4-dichlorophenylamino)thiazolo[5,4-f]quinazoline-2-carbimidate* (**8d**). Prepared from carbonitrile **5d**. Flash chromatography eluent (DCM-EtOAc, 5:5). Yield: 45%; yellow solid; mp = 228–230 °C. IR (cm^−1^) ν*_max_* 3296, 2920, 1640, 1608, 1588, 1551, 1507, 1491, 1469, 1437, 1397, 1356, 1284, 1158, 1129, 1073, 1023, 942, 860, 821; ^1^H-NMR (300 MHz, DMSO-*d*_6_) δ 8.42 (d, 1H, *J* = 9 Hz), 8.19 (s, 1H), 7.69 (d, 1H, *J* = 9 Hz), 7.56 (d, 2H, *J* = 9 Hz), 7.22 (m, 1H), 3.95 (s, 3H); HRMS calcd for C_17_H_12_N_5_OSCl_2_ [M + H]^+^: 404.0140, found 404.0135.

*Methyl 9-(4-fluorophenylamino)thiazolo[5,4-f]quinazoline-2-carbimidate* (**8e**). Prepared from carbonitrile **5e**. Flash chromatography eluent (EtOAc). Yield: 77%; yellow solid; mp > 260 °C. IR (cm^−1^) ν*_max_* 3416, 3298, 3226, 3150, 2950, 1731, 1641, 1611, 1574, 1558, 1506, 1490, 1434, 1400, 1355, 1329, 1285, 1226, 1157, 1103, 1072, 994, 968, 943, 819; ^19^F-NMR (282 MHz, DMSO-*d*_6_) δ −120.8; ^1^H-NMR (300 MHz, DMSO-*d*_6_) δ 9.33 (s, 1H, NH), 8.41 (d, 1H, *J* = 9.0 Hz), 7.93 (s, 1H), 7.68 (d, 1H, *J* = 9.0 Hz), 7.19 (m, 4H), 3.95 (s, 3H); HRMS calcd for C_17_H_13_N_5_OSF [M + H]^+^: 354.0825, found 354.0811.

*Methyl 9-(4-bromo-2-fluorophenylamino)thiazolo[5,4-f]quinazoline-2-carbimidate* (**8f**) and *methyl 9-(3-chloro-4-fluorophenylamino)thiazolo[5,4-f]quinazoline-2-carbimidate* (**8i**) were synthesized in Reference [9].

*Methyl 9-(4-chloro-2-fluorophenylamino)thiazolo[5,4-f]quinazoline-2-carbimidate* (**8h**). Prepared from carbonitrile **5h**. Flash chromatography eluent (EtOAc). Yield: 58%; yellow solid; mp > 260 °C. IR (cm^−1^) ν*_max_* 2953, 1641, 1600, 1553, 1507, 1481, 1397, 1355, 1287, 1198, 1159, 1120, 1072, 944, 899, 818; ^19^F-NMR (282 MHz, DMSO-*d*_6_) δ −120.1; ^1^H-NMR (300 MHz, DMSO-*d*_6_) δ 9.34 (s, 1H, NH), 8.45 (d, 1H, *J* = 9.0 Hz), 8.14 (s, 1H), 7.71 (d, 1H, *J* = 9.0 Hz), 7.44 (d, 1H, *J* = 9.0 Hz), 7.24 (m, 2H), 3.94 (s, 3H); HRMS calcd for C_17_H_12_N_5_OSClF [M + H]^+^: 388.0435, found 388.0426.

*Methyl 9-(2-fluoro-4-methoxyphenylamino)thiazolo[5,4-f]quinazoline-2-carbimidate* (**8i**). Prepared from carbonitrile **5i**. Flash chromatography eluent (EtOAc). Yield: 82%; yellow solid; mp = 224–226 °C. IR (cm^−1^) ν*_max_* 3150, 2950, 1645, 1601, 1570, 1488, 1435, 1355, 1322, 1285, 1269, 1203, 1155, 1096, 1069, 1032, 939, 819; ^19^F-NMR (282 MHz, DMSO-*d*_6_) δ −120.44; ^1^H-NMR (300 MHz, DMSO-*d*_6_) δ 9.33 (s, 1H, NH), 8.42 (d, 1H, *J* = 9.0 Hz), 8.06 (s, 1H), 7.70 (d, 1H, *J* = 9.0 Hz), 7.19 (m, 1H), 6.88 (m, 1H), 6.77 (m, 1H), 3.94 (s, 3H), 3.78 (s, 3H); HRMS calcd for C_18_H_15_N_5_O_2_SF [M + H]^+^: 384.0927, found 384.0930.

*Methyl 9-(3-fluoro-4-hydroxyphenylamino)thiazolo[5,4-f]quinazoline-2-carbimidate* (**8j**). Prepared from carbonitrile **5j**. Flash chromatography eluent (EtOAc). Yield: 70%; pale brown solid; mp > 260 °C. IR (cm^−1^) ν*_max_* 3374, 1729, 1652, 1626, 1585, 1519, 1465, 1386, 1352, 1302, 1241, 1209, 1156, 1111, 978, 856, 827; ^19^F-NMR (282 MHz, DMSO-*d*_6_) δ −138.5; ^1^H-NMR (300 MHz, DMSO-*d*_6_) δ 8.52 (d, 1H, *J* = 9.0 Hz), 8.19 (s, 1H), 7.72 (d, 1H, *J* = 9.0 Hz), 7.04–6.91 (m, 3H), 3.95 (s, 3H); HRMS calcd for C_17_H_13_N_5_O_2_SF [M + H]^+^: 370.0774, found 370.0762.

*Methyl 9-(2,4-difluorophenylamino)thiazolo[5,4-f]quinazoline-2-carbimidate* (**8k**). Prepared from carbonitrile **5k**. Flash chromatography eluent (EtOAc). Yield: 71%; yellow solid; mp > 260 °C; IR (cm^−1^) ν*_max_* 1644, 1608, 1574, 1556, 1509, 1488, 1435, 1357, 1285, 1260, 1188, 1140, 1073, 963, 943, 843, 819; ^19^F-NMR (282 MHz, DMSO-*d*_6_) δ −117.6, −118.8; ^1^H-NMR (300 MHz, DMSO-*d*_6_) δ 9.34 (s, 1H, NH), 8.45 (d, 1H, *J* = 9.0 Hz), 8.12 (s, 1H), 7.70 (d, 1H, *J* = 9.0 Hz), 7.27 (m, 2H), 7.05 (t, 1H, *J* = 7.8 Hz), 3.94 (s, 3H); HRMS calcd for C_17_H_12_N_5_OSF_2_ [M + H]^+^: 372.0731, found 372.0725.

*Methyl 9-(4-(trifluoromethyl)phenylamino)thiazolo[5,4-f]quinazoline-2-carbimidate* (**8l**). Prepared from carbonitrile **5l**. Flash chromatography eluent (EtOAc). Yield: 53%; pale yellow solid; mp > 260 °C. IR (cm^−1^) ν*_max_* 3277, 1643, 1601, 1588, 1561, 1509, 1493, 1324, 1284, 1151, 1104, 1065, 1015, 966, 937, 829, 809; ^19^F-NMR (282 MHz, DMSO-*d*_6_) δ −59.95; ^1^H-NMR (300 MHz, DMSO-*d*_6_) δ 8.42 (d, 1H, *J* = 9.0 Hz), 8.11 (s, 1H), 7.69 (d, 3H, *J* = 7.8 Hz), 7.32 (d, 2H, J = 7.8 Hz), 3.93 (s, 3H); HRMS calcd for C_18_H_13_N_5_OSF_3_ [M + H]^+^: 404.0736, found 404.0742.

Series **9a**–**h**: Compounds Bearing ^9^*N*-Phenyl Groups with Alkyl, Amines and Nitrogen Containing Substituents

*Methyl 9-(phenylamino)thiazolo[5,4-f]quinazoline-2-carbimidate* (**9a**). Prepared from carbonitrile **6a**. Flash chromatography eluent (EtOAc). Yield: 52%; yellow solid; mp > 260 °C. IR (cm^−1^) ν*_max_* 3294, 3147, 2950, 2877, 1727, 1640, 1609, 1570, 1552, 1507, 1480, 1434, 1351, 1284, 1210, 1153, 1067, 990, 965, 939, 869, 819; ^1^H-NMR (300 MHz, DMSO-*d*_6_) δ 9.32 (s, 1H, NH), 8.41 (d, 1H, *J* = 9.0 Hz), 7.99 (s, 1H), 7.68 (d, 1H, *J* = 9.0 Hz), 7.38 (m, 2H), 7.09 (m, 3H), 3.94 (s, 3H); HRMS calcd for C_17_H_14_N_5_OS [M + H]^+^: 336.0919, found 336.0904.

*Methyl 9-(p-tolylamino)thiazolo[5,4-f]quinazoline-2-carbimidate* (**9b**). Prepared from carbonitrile **6b**. Flash chromatography eluent (EtOAc). Yield: 88%; yellow solid; mp > 260 °C. IR (cm^−1^) ν*_max_* 3292, 3148, 2848, 1725, 1641, 1600, 1557, 1488, 1434, 1351, 1283, 1155, 1068, 990, 966, 939, 820, 804; ^1^H-NMR (300 MHz, DMSO-*d*_6_) δ 9.33 (s, 1H, NH), 8.41 (d, 1H, *J* = 9.0 Hz), 7.95 (s, 1H), 7.69 (d, 1H, *J* = 9.0 Hz), 7.20 (m, 2H), 7.11 (m, 2H), 3.94 (s, 3H), 2.32 (s, 3H); HRMS calcd for C_18_H_16_N_5_OS [M + H]^+^: 350.1076, found 350.1072.

Methyl 9-(4-*tert*-butylphenylamino)thiazolo*[5,4-f]*quinazoline-2-carbimidate (**9d**). Prepared from carbonitrile **6c**. Flash chromatography eluent (EtOAc). Yield: 69%; yellow solid; ; mp > 260 °C. IR (cm^−1^) ν*_max_* 3267, 2939, 1731, 1644, 1599, 1580, 1493, 1342, 1269, 1248, 1161, 1114, 1066, 988, 965, 941, 899, 838; ^1^H-NMR (300 MHz, DMSO-*d*_6_) δ 9.31 (s, 1H, NH), 8.39 (d, 1H, *J* = 9.0 Hz), 8.17 (s, 1H), 7.84 (d, 1H, *J* = 9.0 Hz), 7.41 (d, 2H, *J* = 7.8 Hz), 7.12 (m, 2H), 3.93 (s, 3H), 1.31 (s, 9H); HRMS calcd for C_22_H_22_N_5_OS (M + H^+^): 392.1545, found 392.1539.

*Methyl 9-(3-ethynylphenylamino)thiazolo[5,4-f]quinazoline-2-carbimidate* (**9d**). Prepared from carbonitrile **6d**. Flash chromatography eluent (EtOAc). Yield: 68%; yellow solid; mp = 220–222 °C. IR (cm^−1^) ν*_max_* 3293, 2950, 1731, 1644, 1613, 1552, 1489, 1437, 1353, 1286, 1157, 1070, 968, 941, 871, 822; ^1^H-NMR (300 MHz, DMSO-*d*_6_) δ 8.43 (d, 1H, *J* = 9 Hz), 8.11 (s, 1H), 7.70 (d, 1H, *J* = 9 Hz), 7.37 (t, 1H, *J* = 7.8 Hz), 7.26–7.16 (m, 3H), 4.16 (s, 1H), 3.95 (s, 3H); HRMS calcd for C_19_H_14_N_5_OS [M + H]^+^: 360.0919, found 360.0908.

*Methyl 9-(4-cyanophenylamino)thiazolo[5,4-f]quinazoline-2-carbimidate* (**9e**). Prepared from carbonitrile **6e**. Flash chromatography eluent (EtOAc). Yield: 52%; yellow solid; mp > 260 °C. IR (cm^−1^) ν*_max_* 3264, 2215, 1655, 1625, 1591, 1561, 1493, 1435, 1385, 1335, 1272, 1227, 1146, 1069, 995, 938, 848, 815; ^1^H-NMR (300 MHz, DMSO-*d*_6_) δ 9.32 (s, 1H, NH), 8.43 (d, 1H, *J* = 9.0 Hz), 8.17 (s, 1H), 7.79 (d, 2H, *J* = 6.9 Hz), 7.68 (d, 1H, *J* = 7.2 Hz), 7.32 (d, 2H, *J* = 6.9 Hz), 3.94 (s, 3H); HRMS calcd for C_18_H_13_N_6_OS [M + H]^+^: 361.0872, found 361.0863.

*Methyl 9-(3-cyanophenylamino)thiazolo[5,4-f]quinazoline-2-carbimidate* (**9f**). Prepared from carbonitrile **6f**. Flash chromatography eluent (EtOAc). Yield: 33%; pale yellow solid; mp > 260 °C. IR (cm^−1^) ν*_max_* 3272, 2235, 1722, 1638, 1615, 1581, 1571, 1491, 1437, 1143, 1109, 1067, 989, 968, 943, 885, 840; ^1^H-NMR (300 MHz, DMSO-*d*_6_) δ 9.34 (s, 1H, NH), 8.45 (d, 1H, *J* = 9.0 Hz), 8.20 (s, 1H), 7.68 (d, 1H, *J* = 9.0 Hz), 7.61 (m, 4H), 3.95 (s, 3H); HRMS calcd for C_18_H_13_N_6_OS [M + H]^+^: 361.0872, found 361.0862.

*Methyl 9-(1H-benzo[d]imidazol-6-ylamino)thiazolo[5,4-f]quinazoline-2-carbimidate* (**9g**). Prepared from carbonitrile **6g**. Flash chromatography eluent (DCM-MeOH, 8:2). Yield: 57%; yellow solid; mp > 260 °C. IR (cm^−1^) ν*_max_* 3094, 1641, 1615, 1573, 1479, 1380, 1343, 1294, 1199, 1141, 1070, 947, 824; ^1^H-NMR (300 MHz, DMSO-*d*_6_) δ 8.46–8.36 (m, 2H), 8.18 (m, 1H), 8.02 (d, 1H, *J* = 9 Hz), 7.73 (d, 1H, *J* = 8.1 Hz), 7.65 (d, 1H, *J* = 8.1 Hz), 7.54 (m, 1H), 3.95 (s, 3H); HRMS calcd for C_18_H_14_N_7_OS [M + H]^+^: 376.0981, found 376.0974.

*Methyl 9-(4-(dimethylamino)phenylamino)thiazolo[5,4-f]quinazoline-2-carbimidate* (**9h**). Prepared from carbonitrile **6h**. Flash chromatography eluent (EtOAc). Yield: 94%; beige solid; mp > 260 °C. IR (cm^−1^) ν*_max_* 3288, 2945, 1629, 1608, 1577, 1520, 1496, 1444, 1337, 1291, 1275, 1209, 1183, 1167, 1068, 968, 943, 820; ^1^H-NMR (300 MHz, DMSO-*d*_6_) δ 9.35 (s, 1H, NH), 8.42 (d, 1H, *J* = 9.0 Hz), 8.07 (s, 1H), 7.77 (d, 1H, *J* = 9.0 Hz), 7.36 (d, 2H, *J* = 8.4 Hz), 6.91 (d, 2H, *J* = 8.4 Hz), 3.94 (s, 3H), 2.99 (s, 3H); HRMS calcd for C_19_H_19_N_6_OS [M + H]^+^: 379.1341, found 379.1330.

*Methyl 9-(4-(pyrrolidin-1-yl)phenylamino)thiazolo[5,4-f]quinazoline-2-carbimidate* (**9i**). Prepared from carbonitrile **6i**. Flash chromatography eluent (DCM-EtOAc, 5:5). Yield: 75%; beige solid; mp > 260 °C. IR (cm^−1^) ν*_max_* 3293, 2847, 1632, 1608, 1577, 1520, 1491, 1444, 1388, 1293, 1265, 1209, 1178, 1163, 1107, 1062, 968, 927, 820, 806; ^1^H-NMR (300 MHz, DMSO-*d*_6_) δ 9.35 (s, 1H, NH), 8.42 (d, 1H, *J* = 9.0 Hz), 8.06 (s, 1H), 7.77 (d, 1H, *J* = 9.0 Hz), 7.34 (d, 2H, *J* = 8.4 Hz), 6.72 (d, 2H, *J* = 8.4 Hz), 3.94 (s, 3H), 3.29 (m, 4H), 1.98 (m, 4H); HRMS calcd for C_21_H_21_N_6_OS [M + H]^+^: 405.1457, found 405.1452.

#### 3.2.3. SAR Studies

Synthesis of ^9^*N*-Methylated-thiazolo[5,4-*f*]quinazoline-2-carbonitriles (**10a–c**)

Methyl iodide (0.90 mmol) was added dropwise to a stirred suspension of carbonitrile **4a**, **4c** and **4e** (0.60 mmol) and sodium hydride (0.90 mmol, 60% dispersion in mineral oil) in dimethylformamide (4 mL). The mixture was stirred for 1 h at 0 °C and then for 2 h at room temperature. After cooling, the resulting mixture was concentrated under reduced pressure. The crude residue obtained was purified by flash chromatography (DCM-ethyl acetate, 1:9) to give **10a**–**c**.

*9-[(4-Methoxyphenyl)(methyl)amino]thiazolo[5,4-f]quinazoline-2-carbonitrile* (**10a**). Prepared from carbonitrile **4a**. Flash chromatography eluent (EtOAc). Yield: 60%; orange solid; mp > 260 °C. IR (cm^−1^) ν*_max_* 3073, 2949, 2908, 2835, 2225, 1615, 1551, 1497, 1481, 1461, 1452, 1436, 1362, 1235, 1153, 1060, 1031, 974, 839, 825, 801; ^1^H-NMR (300 MHz, DMSO-*d*_6_) δ 8.49 (d, 1H, *J* = 9.0 Hz), 8.23 (s, 1H), 7.88 (d, 1H, *J* = 9.0 Hz), 7.48 (d, 1H, *J* = 8.7 Hz), 6.92 (d, 1H, *J* = 8.7 Hz), 3.77 (s, 3H), 3.73 (s, 3H); HRMS calcd for C_18_H_14_N_5_OS [M + H]^+^: 348.0919, found 348.0908.

*9-[(3,4-Dimethoxyphenyl)(methyl)amino]thiazolo[5,4-f]quinazoline-2-carbonitrile* (**10b**). Prepared from carbonitrile **4e**. Flash chromatography eluent (EtOAc). Yield: 74%; orange solid; mp = 222–224 °C. IR (cm^−1^) ν*_max_* 3040, 2988, 2957, 2828, 2225, 1621, 1553, 1501, 1442, 1409, 1392, 1366, 1255, 1227, 1201, 1173, 1142, 1123, 1023, 936, 872, 803; ^1^H-NMR (300 MHz, DMSO-*d*_6_) δ 8.56 (d, 1H, *J* = 9.0 Hz), 8.29 (s, 1H), 7.85 (d, 1H, *J* = 9.0 Hz), 7.17 (d, 1H, *J* = 2.1 Hz), 7.08 (dd, 1H, *J*_1_ = 2.1 Hz, *J*_2_ = 8.7 Hz), 6.93 (d, 1H, *J* = 8.7 Hz), 3.77 (m, 9H); HRMS calcd for C_19_H_16_N_5_O_2_S [M + H]^+^: 378.1025, found 378.1008.

*9-[(2,3-Dihydrobenzo[b][1,4]dioxin-6-yl)(methyl)amino]thiazolo[5,4-f]quinazoline-2-carbonitrile* (**10c**). Prepared from carbonitrile **4c**. Flash chromatography eluent (EtOAc). Yield: 30%; orange solid; mp > 260 °C. IR (KBr) ν*_max_*/cm^−1^ 3422, 2932, 2875, 2220, 1612, 1547, 1487, 1455, 1360, 1299, 1253, 1201, 1149, 1065, 914, 877, 811. ^1^H-NMR (300 MHz, DMSO-*d*_6_) δ 8.54 (d, 1H, *J* = 9.0 Hz), 8.28 (s, 1H), 7.84 (d, 1H, *J* = 9.0 Hz), 7.12 (d, 1H, *J* = 2.4 Hz), 6.98 (dd, 1H, *J*_1_ = 2.4 Hz, *J*_2_ = 8.7 Hz), 6.82 (d, 1H, *J* = 8.7 Hz), 4.24 (s, 4H), 3.76 (s, 3H); HRMS calcd for C_19_H_13_N_5_O_2_S [M + H]^+^: 375.0819, found 375.0808.

Synthesis of Methyl Thiazolo[5,4-*f*]quinazoline-2-carbimidates (**11a**, **11b** and **11c**)

A stirred mixture of carbonitrile **10a**, **10b** or **10c** (0.13 mmol) and NaOCH_3_ (0.5 M sol. in MeOH, 130 μL) in methanol (4 mL) was irradiated under microwaves at 65 °C for 30 min. The solvent was removed *in vacuo* and the crude residue purified by flash chromatography to afford imidates **11a**, **11b** and **11c**, respectively.

*Methyl 9-[(4-methoxyphenyl)(methyl)amino]thiazolo[5,4-f]quinazoline-2-carbimidate* (**11a**). Prepared from carbonitrile **10a**. Flash chromatography eluent (DCM-MeOH, 9:1). Yield: 93%; yellow solid; mp = 246–248 °C. IR (cm^−1^) ν*_max_* 3267, 3057, 2929, 2837, 1736, 1654, 1613, 1555, 1493, 1434, 1404, 1369, 1330, 1268, 1240, 1219, 1146, 1100, 1058, 1033, 982, 939, 886, 835, 811; ^1^H-NMR (300 MHz, MeOD-*d_4_*) δ 8.42 (d, 1H, *J* = 9.0 Hz), 8.18 (s, 1H), 7.71 (d, 1H, *J* = 9.0 Hz), 7.34 (d, 2H, *J* = 9.0 Hz), 6.91 (d, 2H, *J* = 9.0 Hz), 3.96 (s, 3H), 3.75 (s, 3H), 3.73 (s, 3H); HRMS calcd for C_19_H_18_N_5_O_2_S [M + H]^+^: 380.1181, found 380.1179.

*Methyl 9-[(3,4-dimethoxyphenyl)(methyl)amino]thiazolo[5,4-f]quinazoline-2-carbimidate* (**11b**). Prepared from carbonitrile **10b**. Flash chromatography eluent (DCM-MeOH, 9:1). Yield: 73%; yellow solid; mp = 220–222 °C. IR (cm^−1^) ν*_max_* 3298, 2986, 2832, 1644, 1619, 1555, 1492, 1434, 1361, 1253, 1228, 1162, 1142, 1127, 1069, 1026, 953, 927, 832, 800; ^1^H-NMR (300 MHz, DMSO-*d*_6_) δ 8.46 (d, 1H, *J* = 9 Hz), 8.21 (s, 1H), 7.75 (d, 1H, *J* = 9 Hz), 7.03 (d, 1H, *J* = 2.1 Hz), 6.93 (d, 1H, *J* = 8.4 Hz), 6.84 (dd, 1H, *J*_1_ = 2.1 Hz, *J*_2_ = 8.4 Hz), 3.96 (s, 3H), 3.75 (s, 9H); HRMS calcd for C_20_H_19_N_5_O_3_S [M + H]^+^: 409.1230, found 409.1219.

*Methyl 9-[(2,3-dihydrobenzo[b][1,4]dioxin-6-yl)(methyl)amino]thiazolo[5,4-f]quinazoline-2-carbimidate* (**11c**). Prepared from carbonitrile **10c**. Flash chromatography eluent (DCM-MeOH, 9:1). Yield: 66%; yellow solid; mp = 248–250 °C. IR (cm^−1^) ν*_max_* 3298, 2973, 2875, 1642, 1620, 1555, 1484, 1435, 1408, 1360, 1298, 1273, 1242, 1203, 1162, 1146, 1065, 939, 878, 847; ^1^H-NMR (300 MHz, DMSO-*d*_6_) δ 8.45 (d, 1H, *J* = 9 Hz), 8.21 (s, 1H), 7.74 (d, 1H, *J* = 9 Hz), 6.97 (d, 1H, *J* = 2.1 Hz), 6.83 (m, 2H), 4.24 (s, 4H), 3.96 (s, 3H), 3.75 (s, 3H); HRMS calcd for C_20_H_18_N_5_O_3_S [M + H]^+^: 408.1130, found 408.1111.

Synthesis of Ethyl, Isopropyl and Benzyl Thiazolo[5,4-*f*]quinazoline-2-carbimidates (**12a–c**)

*Ethyl 9-(benzo[d][1,3]dioxol-5-ylamino)thiazolo[5,4-f]quinazoline-2-carbimidate* (**12a**). A stirred mixture of carbonitrile **4b** (0.05 g, 0.14 mmol) and NaOCH_2_CH_3_ (0.5 M sol. in EtOH, 130 μL) in ethanol (4 mL) was heated under microwaves (600 W) at 80 °C for 30 min. The solvent was removed *in vacuo* and the crude residue purified by flash chromatography (DCM-EtOAc, 5:5) to afford ethyl imidate **12a** (0.043 g 79%) as a yellow solid; mp = 193–195 °C. IR (cm^−1^) ν*_max_* 3286, 2892, 1722, 1654, 1626, 1579, 1497, 1484, 1465, 1372, 1334, 1242, 1230, 1184, 1159, 1128, 1036, 966, 923, 824; ^1^H-NMR (300 MHz, DMSO-*d*_6_) δ 8.39 (d, 1H, *J* = 9.0 Hz), 7.94 (s, 1H), 7.68 (d, 1H, *J* = 9.0 Hz), 6.94 (d, 1H, *J* = 8.1 Hz), 6.76–6.46 (m, 2H), 6.01 (s, 2H), 4.38 (q, 2H, *J* = 6.9 Hz), 1.38 (t, 3H, *J* = 6.9 Hz); HRMS calcd for C_19_H_16_N_5_O_3_S [M + H]^+^: 394.0974, found 394.0967.

*Isopropyl 9-(benzo[d][1,3]dioxol-5-ylamino)thiazolo[5,4-f]quinazoline-2-carbimidate* (**12b**). A stirred mixture of carbonitrile **4b** (0.078 g, 0.22 mmol) and KOH (2.5 N sol., 78 μL) in isopropanol (3.9 mL) was heated under microwaves (600 W) at 100 °C for 2 h. The solvent was removed *in vacuo* and the crude residue purified by flash chromatography (DCM-EtOAc, 5:5) to afford the isopropyl imidate **12b** (0.024 g, 27%) as a yellow solid, mp = 224–226 °C. IR (cm^−1^) ν*_max_* 3267, 2977, 2876, 1638, 1613, 1572, 1489, 1475, 1450, 1382, 1369, 1317, 1272, 1244, 1189, 1142, 1112, 1036, 924, 885, 828, 808; ^1^H-NMR (300 MHz, DMSO-*d*_6_) δ 8.40 (d, 1H, *J* = 9.0 Hz), 7.94 (s, 1H), 7.68 (d, 1H, *J* = 9.0 Hz), 6.94 (d, 2H, *J* = 8.1 Hz), 6.75–6.55 (m, 2H), 6.01 (s, 2H), 5.32–5.24 (m, 1H), 1.38 (d, 6H, *J* = 6.0 Hz); HRMS calcd for C_20_H_18_N_5_O_3_S [M + H]^+^: 408.0962, found 408.0956.

*Benzyl 9-(benzo[d][1,3]dioxol-5-ylamino)thiazolo[5,4-f]quinazoline-2-carbimidate* (**12c**). A stirred mixture of carbonitrile **4b** (0.05 g, 0.14 mmol) and NaOCH_2_Ph (1.0 M sol. in benzylalcohol, 70 μL) in benzylalcohol (3 mL) was irradiated under microwaves at 100 °C for 30 min. The solvent was removed *in vacuo* and the crude residue purified by flash chromatography (EtOAc) to afford the benzyl imidate **12c** (0.018 g, 28%) as a yellow solid, mp = 180–182 °C. IR (cm^−1^) ν*_max_* 3375, 2228, 1726, 1644, 1613, 1575, 1473, 1378, 1327, 1244, 1192, 1151, 1036, 922, 833; ^1^H-NMR (300 MHz, DMSO-*d*_6_) δ 8.42 (d, 1H, *J* = 9.0 Hz), 7.99 (s, 1H), 7.68 (d, 1H, *J* = 9.0 Hz), 7.51 (d, 2H, *J* = 7.5 Hz), 7.43–7.34 (m, 3H), 6.92 (d, 1H, *J* = 7.5 Hz), 6.78 (m, 1H), 6.59 (m, 1H), 6.01 (s, 2H), 5.45 (s, 2H); HRMS calcd for C_24_H_18_N_5_O_3_S [M + H]^+^: 456.1130, found 456.1128.

*Methyl 9-(benzo[d][1,3]dioxol-5-ylamino)thiazolo[5,4-f]quinazoline-2-carboxylate* (**13**). A mixture of methyl 9-(benzo[*d*][1,3]dioxol-5-ylamino)thiazolo[5,4-f]quinazoline-2-carbimidate (**7b**) (0.017 mmol) and 5 mL of MeOH/H_2_O + TFA(0.1%)(60/40) under argon was stirred at room temperature overnight. The solvent was removed *in vacuo* and the crude residue purified by flash chromatography (DCM-EtOAc, 5:5) to afford ester **13** (5.9 mg, 94% yield) as a yellow solid; mp = 206 °C. IR (cm^−1^) ν*_max_* 3287, 2902, 1648, 1617, 1575, 1528, 1499, 1483, 1452, 1432, 1385, 1322, 1272, 1196, 1125, 1043, 936, 885, 834, 817; ^1^H-NMR (300 MHz, DMSO-*d*_6_) δ 8.42 (d, 1H, *J* = 9.0 Hz), 8.03 (s, 1H), 7.95 (d, 1H, *J* = 9.0 Hz), 6.96 (d, 1H, *J* = 8.0 Hz), 6.84 (m, 1H), 6.72 (d, 1H, *J* = 8.0 Hz), 5.94 (s, 2H), 4.05 (s, 3H); HRMS calcd for C_18_H_13_N_4_O_4_S [M + H]^+^: 381.0658, found 381.0651.

### 3.3. In Vitro Kinase Preparation and Assays

The DYRK1A and DYRK1B kinase assays to determine IC_50_ values were performed by Reaction Biology Corporation using HotSpot technology [38]. Kinase reaction with specific kinase/substrate pair along with required cofactors was carried out in 20 mM Hepes pH 7.5, 10 mM MgCl_2_, 1 mM EGTA, 0.02% Brij35, 0.02 mg/mL BSA, 0.1 mM Na_3_VO_4_, 2 mM DTT, 1% DMSO. Purified recombinant kinase was incubated with serial 3-fold dilutions of test compounds starting at a final concentration of 10 μM. Reaction was initiated by addition of a mixture of ATP (Sigma, St. Louis, MO, USA) and ^33^P ATP (Perkin Elmer, Waltham, MA, USA) to a final concentration of 10 µM and was carried out at room temperature for 120 min, followed by spotting of the reaction onto P81 ion exchange filter paper (Whatman Inc., Piscataway, NJ, USA). Unbound phosphate was removed by extensive washing of filters in 0.75% Phosphoric acid. After subtraction of background derived from control reactions containing inactive enzyme, kinase activity data was expressed as the percent of remaining kinase activity in test samples compared to vehicle (DMSO) reactions. Dose response curves were fitted using Prism 5.0 from Graph-Pad Software.

## 4. Conclusions

The convenient synthesis of a focused library (forty molecules) of novel 6,6,5-tricyclic thiazolo[5,4-*f*]quinazolines was realized under microwaves using Dimroth rearrangement for construction of the pyrimidine part. A novel 6-aminobenzo[*d*]thiazole-2,7-dicarbonitrile (**1**) was used as a very powerful molecular platform for the synthesis of various thiazolo[5,4-*f*]quinazoline derivatives. On chemical and practical aspects this article is a further example illustrating how microwave heating can be a very powerful tool for medicinal chemistry. The inhibitory potency of the final compounds was evaluated against a panel of two kinases (DYRK1A and DYRK1B). In our screening efforts to discover new scaffolds for the inhibition of DYRK1A, we identified a series of new thiazolo[5,4-*f*]quinazolines that were potent dual DYRK1A/1B inhibitors. Five lead compounds EHT 5372 (**8c**), EHT 1610 (**8i**), EHT 9851 (**8k**), EHT 3356 (**9b**) and EHT 6840 (**8h**) displayed single-digit nanomolar or subnanomolar DYRK1A/1B IC_50_ values and are among the most potent dual DYRK1A/1B inhibitors disclosed to date. Studies to rationalize the SAR observed and to identify the DYRK binding mode with these inhibitors were realized and will be reported in due course. Finally more about the biochemical and biological characterization in different therapeutic areas of these promising lead DYRK1A/1B inhibitors is also in progress and will be reported elsewhere. 
